# Mechanistic Insights in Acceptorless Dehydrogenation of N‑Heterocycles Using Graphenes as Carbocatalysts

**DOI:** 10.1021/acsami.5c11895

**Published:** 2025-12-13

**Authors:** Andrés Mollar-Cuni, Pablo García-Aznar, Santiago Martín, German Sastre, Hermenegildo García, Jose A. Mata

**Affiliations:** a 456141 Institute of Advanced Materials (INAM), Universitat Jaume I, Avda. Sos Baynat s/n, 12006 Castellón, Spain; b Instituto de Tecnología Química, Consejo Superior de Investigaciones Científicas—Universitat Politècnica de València, Avda. Los Naranjos s/n, 46022 Valencia, Spain; c Instituto de Nanociencia y Materiales de Aragón (INMA), CSIC—Universidad de Zaragoza, 50009, Zaragoza, Spain; d Departamento de Química Física, Universidad de Zaragoza, 50009 Zaragoza, Spain; e Laboratorio de Microscopias Avanzadas (LMA), 16765 Universidad de Zaragoza, Edificio I+D+i, 50018 Zaragoza, Spain

**Keywords:** carbocatalysis, acceptorless dehydrogenation, graphene, N-heterocycles, reaction mechanism

## Abstract

Catalytic dehydrogenation is a critical transformation in the chemical and energy sectors, particularly for reversible hydrogen storage systems. One of the most promising systems for hydrogen storage is the development of liquid organic hydrogen carriers (LOHCs), which have the potential capacity of storing and releasing hydrogen gas on demand. Catalytic direct dehydrogenation represents a greener, promising method to generate hydrogen in situ from these hydrogen-dense carriers. The catalytic activity of graphene materials as metal-free carbocatalysts in the acceptorless dehydrogenation of N-heterocycles has been explored. Herein, a detailed mechanistic investigation has been conducted through both experimental (stoichiometric and masking experiments) and DFT calculations on the reaction mechanism. The proposed mechanism identifies *o*-quinone groups as the active sites responsible for catalysis, involving the transformation of *o*-quinone groups into epoxide intermediates, which release molecular hydrogen and regenerate the *o*-quinone groups, completing the catalytic cycle. This work provides insight into the design of efficient metal-free catalysts for their use in LOHCs storage systems, paving the way for sustainable energy solutions.

## Graphical abstract

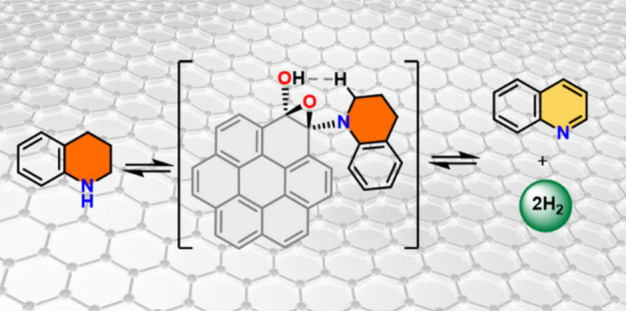

## Introduction

The use of graphenes has experienced an exponential growth in the search of metal-free carbocatalysts.^1–3^ The actual scenario, in the field of catalysis, is predominantly based on the use of scarce and expensive metals, a model which is no longer sustainable. Consequently, there is a growing need to develop alternative catalytic systems based on earth-abundant elements. In this context, graphenes are promising candidates as carbocatalysts due to their composition from abundant elements, high specific surface area, and two-dimensional morphology, which exposes all atoms and facilitates efficient mass transfer.^4,5^

A major challenge in the use of carbocatalysts lies in their ill-defined structural nature, which complicates mechanistic studies and hinders the full understanding of their active sites.^6,7^ Identifying the nature and quantity of these active sites is crucial for the rational design of improved catalytic systems through targeted synthetic protocols. Graphene materials have demonstrated catalytic activity in a variety of oxidation, reduction and coupling reactions. Proposed active sites on carbocatalysts include oxygen-containing functional groups,^8–13^ edge- or periphery-associated regions,^14–16^ carbon vacancies,^17,18^ basal plane carbons^19^ and heteroatom dopants.^20–24^ Furthermore, the inherent properties of graphene two-dimensional structure contribute to its catalytic behavior.^25–27^ However, mechanistic studies of graphene materials in acceptorless dehydrogenation reactions remain largely unexplored, presenting a critical gap in our understanding of their catalytic mechanisms.

Acceptorless dehydrogenation refers to the process where molecular hydrogen (H_2_) is released in its gaseous form.^28–31^ This transformation is thermodynamically endergonic and requires the continuous removal of hydrogen from the reaction medium to drive the process forward. Dehydrogenation and hydrogenation reactions are both necessary to implement hydrogen storage technologies, particularly those employing liquid organic hydrogen carriers (LOHCs).^32–34^ In these systems, hydrogen is stored via the formation of covalent C–H bonds which are subsequently activated to release hydrogen as H_2_ gas. Hydrogen storage in the form of LOHCs holds promise for industrial applications aimed at reducing reliance on fossil fuels and advancing renewable energy technologies. It is also of fundamental academic interest, as C–H activation represents a key reaction in modern chemistry, driving innovation in catalytic systems and hydrogen-based energy storage.

Various LOHCs have been explored for hydrogen storage, including cycloalkanes, alcohols, and primary amines. Among these, N-heterocycles have attracted particular attention due to the influence of the heteroatom in facilitating the release of hydrogen.^35–38^ Moreover, heterocyclic products are widely found in pharmaceuticals and biologically active molecules, underscoring their significance beyond hydrogen storage applications. Their unique structural features and functional versatility make them promising candidates for sustainable energy systems while maintaining relevance to broader chemical and biological contexts.

A detailed mechanistic study of graphene materials as carbocatalyst in the acceptorless dehydrogenation of N-heterocycles is presented herein. The use of graphene nanoplatelets allows precise control over critical material properties such as surface area and functional group composition and emphasizes the identification of key parameters that govern catalytic efficiency in carbocatalysts. Based on experimental evidence from techniques such as NMR spectroscopy, mass spectrometry, masking experiments, and model compound studies, a plausible mechanism for the reaction is proposed, involving the participation of oxygen functionalities in the catalytic cycle. This mechanistic understanding is further supported by density functional theory (DFT) calculations, which model the reaction profile on a graphene coronene cluster.

## Results and Discussion

A series of experiments were conducted using graphene nanoplatelets (GNPs) to investigate the reaction mechanism underlying the acceptorless dehydrogenation of N-heterocycles. The objective was to provide experimental evidence that could elucidate this mechanism while expanding the range of carbon-based catalytic materials applicable to dehydrogenation reactions. This work is built upon previous studies involving reduced graphene oxides.^39^

GNPs are carbonaceous materials derived from the direct exfoliation of graphite. In contrast to reduced graphene oxides (rGOs), which are typically synthesized using chemical reducing agents, GNPs generally possess a greater number of graphene layers and fewer surface functional groups. A key advantage of GNPs lies in the ability to systematically control their flake size and specific surface area through tailored synthetic protocols. The initial phase of this study focused on assessing the catalytic performance of GNPs in the acceptorless dehydrogenation of 2-methyltetrahydroquinoline (2-MeTHQ). Several GNP samples with distinct specific surface areas (750, 500, and 300 m^2^/g) were evaluated. Significant differences in catalytic activity were observed among the samples, as illustrated by the reaction profiles (Figure 1a). The GNP sample with the highest surface area (GNP750) exhibited the greatest catalytic activity, achieving 80% conversion within 400 min. In comparison, GNP300 reached only 32% conversion under identical conditions, while the performance of GNP500 was similar to that of rGO. The superior activity of GNP750 highlights its potential as an advanced carbocatalyst for the acceptorless dehydrogenation of N-heterocycles, outperforming previously reported materials.

**Figure 1:**
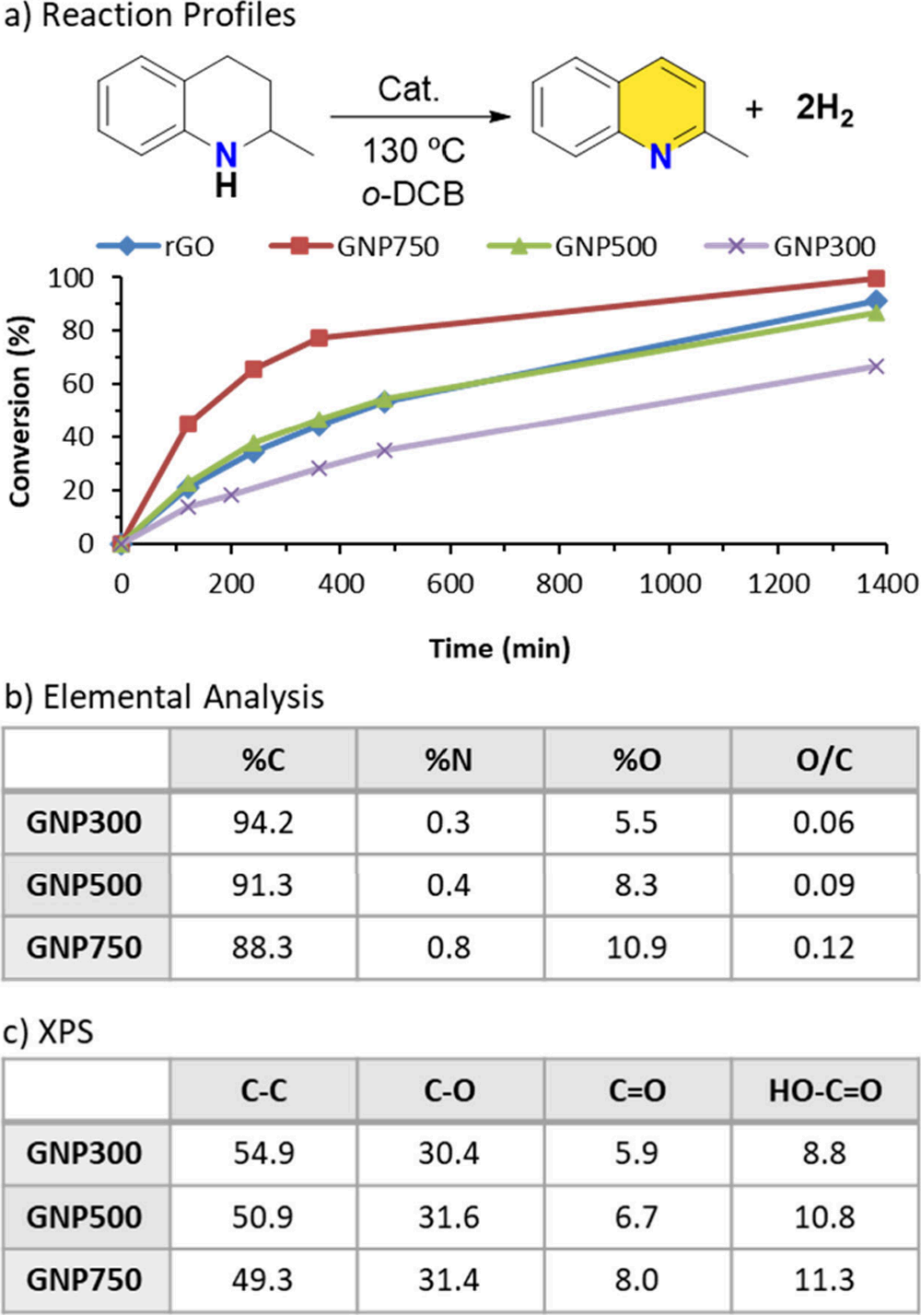
Dehydrogenation of 2-methyltetrahydro­quinoline: (a) reaction profiles obtained using different carbocatalysts. Reaction conditions: 2-MeTHQ (0.15 mmol, 21.5 μL), cat. (15 mg), o-DCB (1 mL), 130 °C oil-bath temperature. (b) Elemental analysis and (c) relative abundance of carbon functional groups (%) determined from Gaussian–Lorentzian fittings of the high-resolution XPS spectra in the C 1s core region.

Attempts to quantify the evolved hydrogen were made using both eudiometric methods and by coupling the reaction setup directly to a micro-GC using standard reaction conditions. However, no reaction occurred under closed-system conditions, indicating that continuous removal of H_2_ is essential to drive this transformation. We observed that acceptorless dehydrogenation proceeds only in reactor systems that allow continuous hydrogen removal to shift the equilibrium, but not when conducted in a sealed environment. Similar behavior was previously reported by Szymczak et al. for the acceptorless dehydrogenation of primary amines.^40^ The acceptorless dehydrogenation of tetrahydroquinolines (or amines) is a thermodynamically uphill process that requires continuous removal of hydrogen from the reaction medium to shift the equilibrium toward product formation. Although quantitative measurement of molecular hydrogen was not possible, qualitative analysis of the reaction headspace confirmed the evolution of molecular hydrogen during the reaction. To facilitate hydrogen detection, the reaction scale was increased 10-fold (Figure S1). In addition, indirect quantification of hydrogen was performed using a trapping experiment. In this approach, the dehydrogenation of THQ was coupled with the hydrogenation of 2-vinylnaphtalene in the presence of comercial Pd/C as catalyst. Gas chromatography analysis revealed quantitative conversion of THQ, while the olefin was hydrogenated with a yield of 79%. Although hydrogenation was not complete, most likely due to hydrogen losses, this trapping experiment provides direct experimental evidence for hydrogen formation and enables an approximate quantification of the evolved hydrogen.

The potential catalytic activity of carbonaceous materials may originate from trace metal impurities. Such impurities can be present in the carbon source or introduced during the preparation protocols. The most common metal impurities found in carbonaceous materials are iron and manganese. To exclude that the catalytic activity arises from these metal impurities, an ICP/MS analysis was performed. The results revealed very low concentrations of manganese (0.0016 wt %) and iron (0.0758 wt %). With these low concentrations and considering our previous findings using rGO in which the addition of these metal impurities in these trace amounts were purposely added without observing any activity change, it is unlikely that the catalytic activity observed in this study is due to residual metal impurities.^41^

To understand the structural and compositional factors underlying the enhanced catalytic activity, a comprehensive characterization of the carbonaceous materials using X-ray photoelectron spectroscopy (XPS), Raman spectroscopy, thermogravimetric analysis (TGA) and transmission electron microscopy (TEM) was performed (Figures S2–S6 and Tables S1–S3). First, the nature of functional groups present in each carbonaceous material was examined through elemental analysis (Figure 1b). The oxygen-to-carbon (O/C) ratio serves as an indicator of the total number of oxygenated groups in the carbon framework. The results suggest a correlation between activity and the abundance of oxygen-containing functional groups. For instance, the most active material, GNP750, exhibited the highest O/C ratio of 0.12, which is significantly greater than the 0.06 ratio observed for GNP300. While elemental analysis provides insight into total oxygen content and the O/C ratio, it does not reveal the specific nature of the oxygenated functional groups. Carbonaceous materials typically contain a variety of such groups, including epoxides, alcohols, ketones, quinones, lactones and carboxylic acids.

To elucidate the specific nature of the oxygen-containing functional groups, XPS analyses were performed (Figures 1c and S2). The results revealed that the portion of C–O single-bonded groups remained constant across all GNPs samples. In contrast, a clear increase in carbonyl (C=O) groups was observed with increasing surface area and catalytic activity. For example, GNP750 exhibited a carbonyl (C=O) content of 8.0%, compared to 5.9% in GNP300. This trend is consistent with the known localization of carbonyl groups at the edges of graphene layers, which become more abundant as surface area increases. Importantly, a linear correlation was established between the carbonyl content and the reaction rate constant (Figure S7). These results suggest that the catalytic activity in acceptorless dehydrogenation of N-heterocycles using GNPs is strongly associated with the presence of carbonyl functional groups.

Using the most active carbonaceous material, GNP750, the substrate scope of the reaction with various N-heterocycles was explored (Figure 2). The results demonstrate that GNP750 is an efficient carbocatalyst for the dehydrogenation of a broad range of 1,2,3,4-tetrahydroquinolines (THQs). Substrates bearing electron-withdrawing groups at the 7-position, such as NO_2_ and CF_3_, showed slightly reduced reactivity, yet still achieved conversions above 75% within 1380 min. In contrast, substitutions at the 6-position—such as OCH_3_, CH_3_, and Cl—led to quantitative conversions within 500 min. To expand the substrate scope, we included additional classes of N-heterocycles beyond substituted THQs. Specifically, we examined tetrahydroisoquinoline, indoline, tetrahydrocarbazole, and tetrahydropyridoindole as substrates. These compounds differ significantly in structure from THQs and were successfully converted to their corresponding dehydrogenated products in good yields. These results highlight the versatility and efficiency of GNP750 as a carbocatalyst for the acceptorless dehydrogenation of N-heterocycles across a diverse set of substrates.

**Figure 2:**
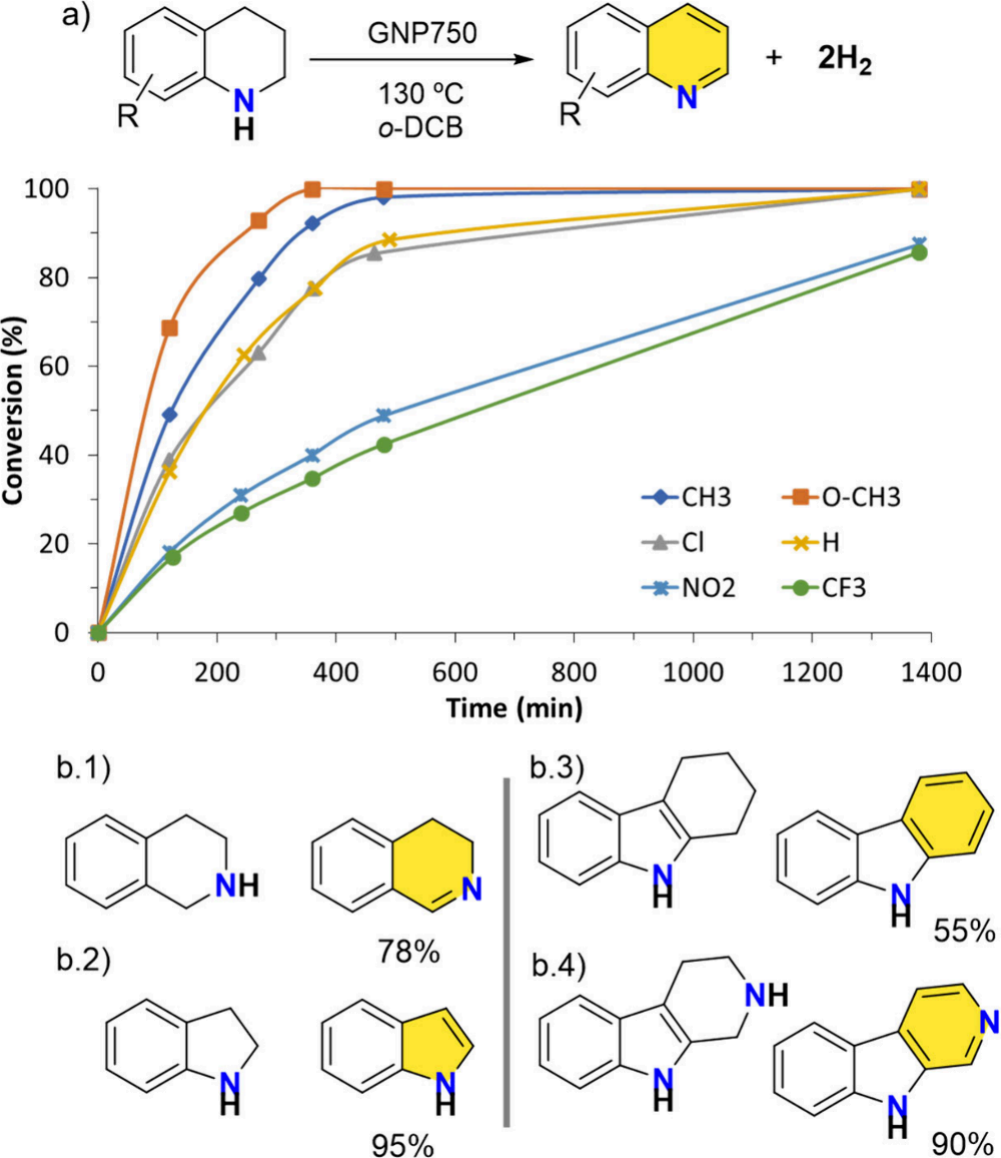
(a) Conversion data for the acceptorless dehydrogenation of 1,2,3,4-THQs using GNP750 as a carbocatalyst. Susbtitution at the 6 position of quinoline scaffold includes R = OCH_3_, CH_3_ and Cl; at the 7 position R = NO_2_ and CF_3_. (b) Dehydrogenation yields for other N-heterocycles. Reaction conditions: substrate (0.15 mmol), GNP-750 (15 mg), o-DCB (1 mL), 130 °C oil-bath temperature.

To gain insight into the activation energy of the dehydrogenation process, reactions were carried out at four different temperatures: 130, 110, 100, and 90 °C over a period of 1400 min (Figure 3). The resulting time–conversion profiles clearly demostrate a temperature-dependent enhancement in catalytic activity. For instance, the yield of 2-MeQ reaches 88% at 130 °C after 480 min but drops to just 38% at 90 °C under the same time frame. By analyzing these data through an Arrhenius plot, the activation energy was calculated to be 42.8 kJ/mol. This value aligns well with previously reported experimental activation energies for the acceptorless dehydrogenation of tetrahydroquinolines using alternative catalysts.^42,43^ Furthermore, these findings are consistent with experimental observations: while the reaction proceeds efficiently at 130 °C, delivering quantitative yields in short times, it also occurs—albeit more slowly—at room temperature, yielding approximately 20% after 72 h.

**Figure 3:**
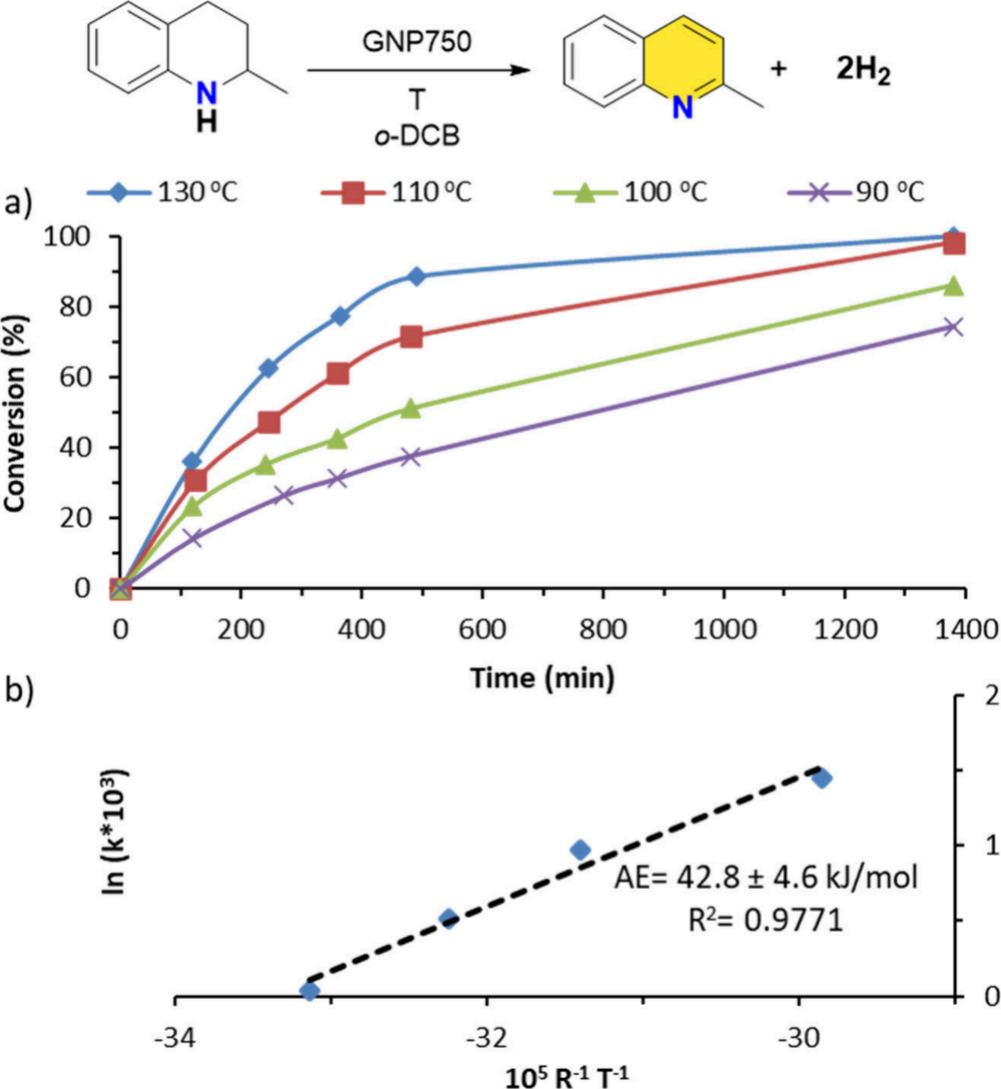
(a) Time–conversion profiles for the dehydrogenation of 2-MeTHQ using GNP750 at various temperatures. Reaction conditions: 2-MeTHQ (0.15 mmol, 21.5 μL), *ortho*-dichlorobenzene (*o*-DCB) (1 mL) and GNP750 (15 mg). (b) Arrhenius plot with linear fit and corresponding equation.

Further mechanistic insights were obtained through masking experiments, a powerful tool for identifying active sites in carbonaceous materials. These experiments are particularly valuable when multiple potential active sites exist, and mass transfer limitations can be excluded. The methodology involves the selective chemical modification of functional groups to generate catalytically inactive derivatives. Suppression of a particular functional group is expected to diminish or eliminate the catalytic activity associated with that site. In previous studies, we employed this strategy to selectively mask ketones, carboxylic acid and hydroxyl groups using phenylhydrazine, 2-bromo-1-phenylethanone and trimethylsilylimidazole, respectively.^39^ This systematic approach enables the isolation of individual functional group contributions to overall catalytic activity, providing critical insights into the specific roles of surface functionalities in carbon-based catalysts.

In our previous work, we reported that the catalytic activity of reduced graphene oxide (rGO) decreased upon treatment with phenylhydrazine, suggesting that carbonyl functionalities (particularly quinone moieties) may serve as active sites in dehydrogenation reactions.^41^ However, the observed suppression of activity was only partial, likely due to incomplete masking of carbonyl groups, leaving some sites accessible. To more selectively mask *o*-quinone-like groups in GNP750, we adopted an alternative masking strategy using ethylenediamine. As a proof of concept, we first examine the reaction between ethylenediamine and 9,10-phenanthrenequinone, a model compound for *o*-quinone masking (Figure 4a). Under basic conditions and using ethanol as solvent at 90 °C, 9,10-phenanthrenequinone was quantitatively converted into a pyrazine derivative (dibenzo­[f,h]­quinoxaline), achieving more than 95% yield (Figure S8). This result confirmed that ethylenediamine is an effective masking agent for *o*-quinones. Subsequently, GNP750 was treated under analogous conditions to afford the masked material GNP750NEtN (Figure 4b). The catalytic activity of this modified material was then evaluated in the dehydrogenation of 2-MeTHQ (Table S4 and Figure S9). In comparison to the parent GNP750 material (Figure 4c), GNP750NEtN exhibited markedly reduced catalytic performance. These results provide strong support for the hypothesis that *o*-quinone groups are the principal oxygen-containing active sites responsible for the catalytic acceptorless dehydrogenation of N-heterocycles.

**Figure 4:**
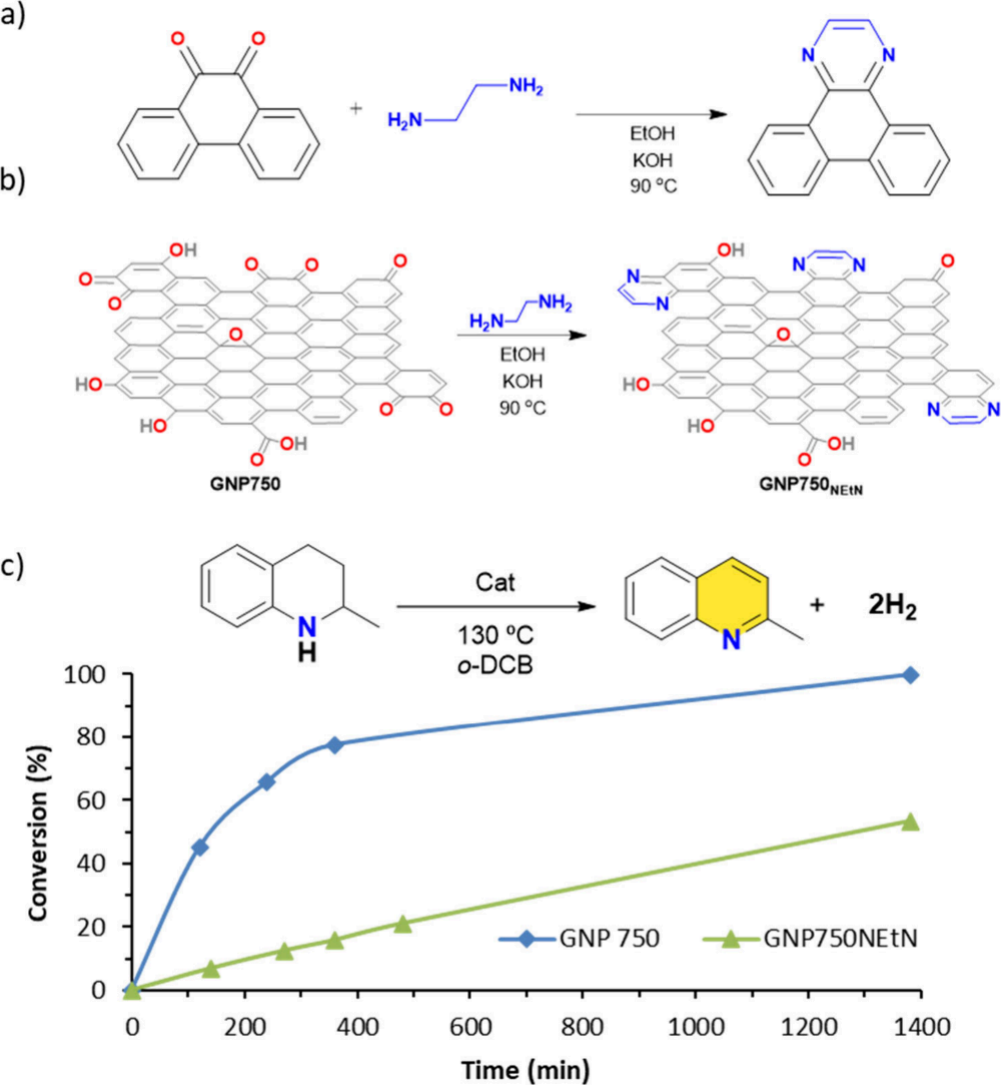
Masking experiments: (a) model reaction between 9,10-phenanthrenequinone and ethylenediamine, (b) schematic representation of ethylenediamine masking of *o*-quinones moieties on GNP750, and (c) comparison of catalytic activity using masked and unmasked GNP750. Reaction conditions: 2-MeTHQ (0.15 mmol, 21.5 μL), cat. (15 mg), o-DCB (1 mL), 130 °C oil-bath temperature.

### Model Molecules as Organocatalysts in the Dehydrogenation of N-Heterocycles

Having established the role of *o*-quinones as key active sites in acceptorless dehydrogenation reaction, we next evaluated model molecules containing quinone functionalities as potential organocatalysts for the dehydrogenation of THQs. Quinone groups have previously been identified as active sites in a range of catalytic transformations, including the reduction of nitrobenzene by hydrazine,^44^ the (photo)­Fenton reaction,^45^ ethylbenzene dehydrogenation,^46,47^ and various oxidative dehydrogenation processes.^48,49^ Drawing from these precedents, we hypothesized that carbonyl groups embedded within polyaromatic frameworks could serve as molecular mimics of the active sites present in carbon-based catalysts, offering a route to define structure–activity relationships and potentially develop metal-free organocatalysts for this transformation.

A series of model molecules with varying in the number of aromatic rings and the presence of carbonyl groups were evaluated as potential organocatalysts for the dehydrogenation of 2-MeTHQ (Figure 5). Compounds lacking carbonyl groups (A) or containing only a single carbonyl group (B) were found to be completely inactive in the dehydrogenation of 2-MeTHQ. In contrast, *ortho*-benzoquinone (C), a simple *o*-quinone species, yielded a modest 11% conversion to 2-methylquinoline (2-MeQ). Notably, significantly improved catalytic performance was observed in systems featuring *o*-quinone moieties embedded within extended polyaromatic frameworks, with yields ranging from 25% to 41%. For instance, pyrene-4,5-dione (D) achieved a 31% yield of 2-MeQ, while the introduction of *tert*-butyl (*t*Bu) groups at the 2,7 positions (E) further enhanced solubility and increrase the yield to 41%. Comparable activity was observed with other polyaromatic *o*-quinone derivatives, such as phenanthroline (1,10-phenanthroline-5,6-dione, F) and phenanthrene (9,10-phenanthrenequinone, G).

**Figure 5:**
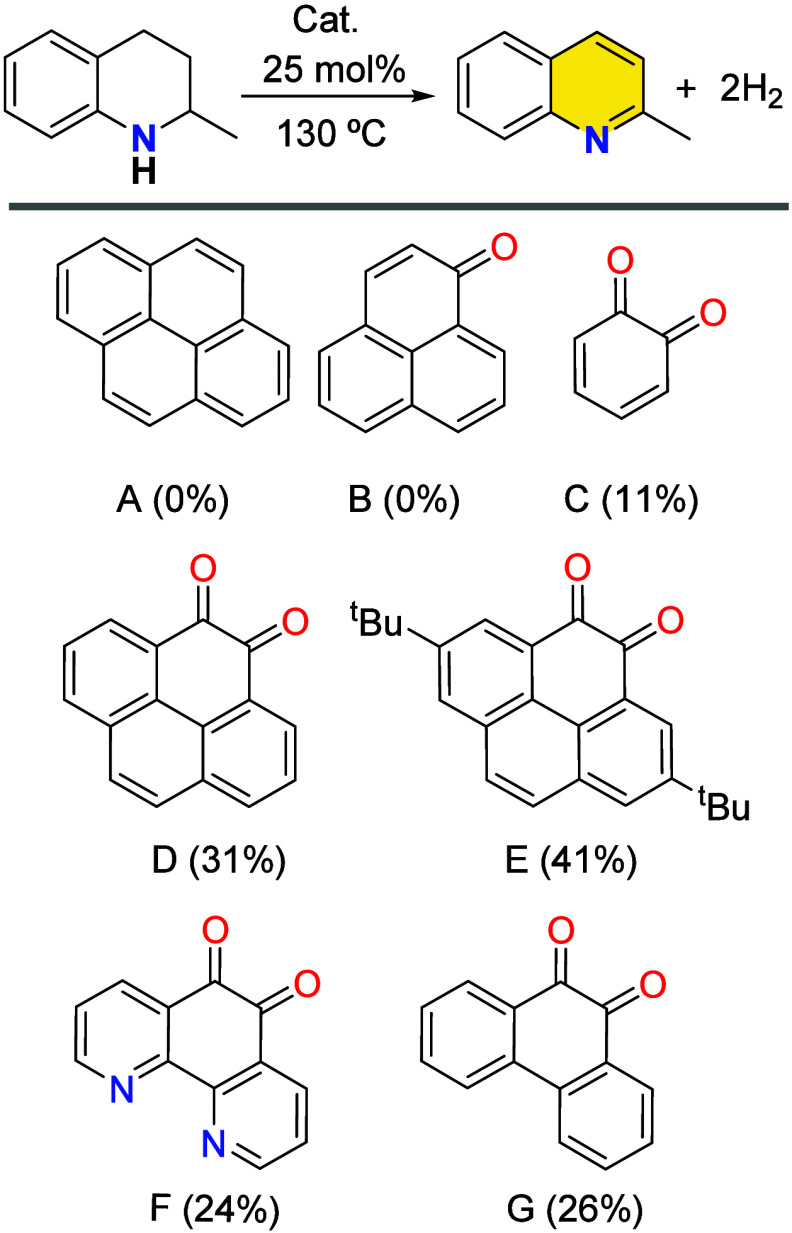
Yields for the acceptorless dehydrogenation of 2-MeTHQ using model molecules as organocatalysts in *o*-DCB (1 mL) over 24 h. Selected organocatalysts are pyrene (A), phenalenone (B), *o*-benzoquinone (C), pyrene-4,5-dione (D), 2,7-bis­(*tert*-butyl)-4,5-pyrenedione (E), 1,10-phenanthroline-5,6-dione (F), 9,10-phenanthrenequinone (G). Reaction conditions: 2-MeTHQ (0.15 mmol, 21.5 μL), cat. (25 mol %, 0.0375 mmol), o-DCB (1 mL), 130 °C oil-bath temperature.

These results underscore the importance of *ortho*-quinone (α, β-dicarbonyl) functional groups and their integration into conjugated sp^2^-hybridized aromatic systems in facilitating the acceptorless dehydrogenation of N-heterocycles. The enhanced performance of these molecular models supports the proposed mechanism involving *o*-quinone sites as the key catalytic motifs in carbon-based dehydrogenation catalysts.

### Initial Steps in Acceptorless Dehydrogenation: Epoxide Formation

The initial steps in the acceptorless dehydrogenation of 2-MeTHQ were studied using stoichiometric amounts of 2,7-Bis­(*tert*-butyl)-4,5-pyrenedione (E) using ortho-dichlorobenzene (o-DCB) as solvent (Figure 6 and Section S9). The reaction was monitored using electrospray ionization mass spectrometry (ESI-MS) to detect potential intermediates. At room temperature, we observed the characteristic peaks for 2-MeTHQ and E as protonated species, labeled [2-MeTHQ + H]^+^ at *m*/*z* 148.2 amu and [E + H]^+^ at *m*/*z* 345.1 amu, respectively. Additionally, the potassium adducts of E appeared at *m*/*z* 383.1 amu, labeled [E + K]^+^.

**Figure 6:**
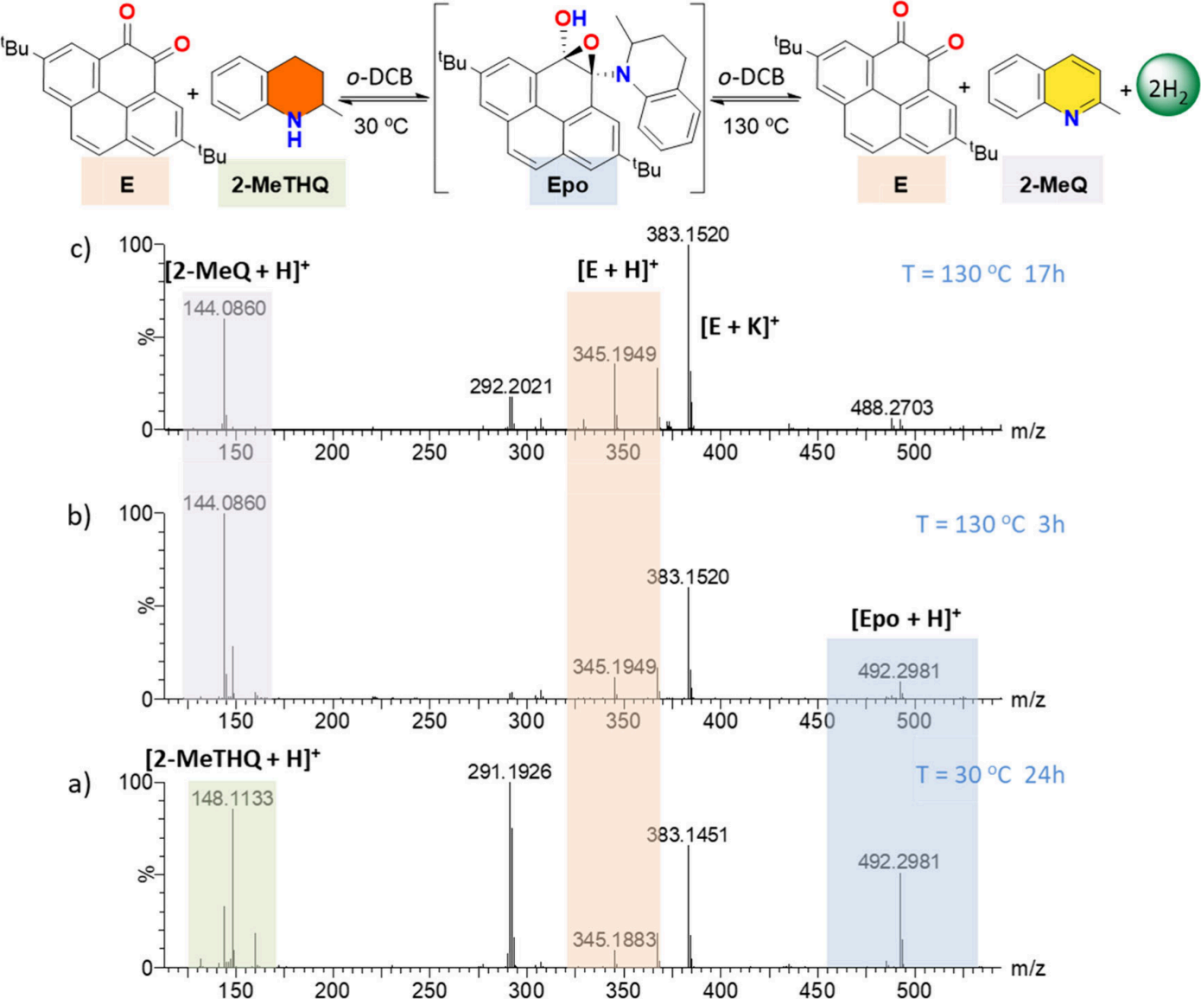
Reaction monitoring during dehydrogenation of 2-MeTHQ using 2,7-bis­(*tert*-butyl)-4,5-pyrenedione (E). ESI mass spectra of the catalytic reaction under different conditions: (a) 24 h at 30 °C, (b) 3 h at 130 °C and (c) 24 h at 130 °C. Samples were diluted with methanol to a concentration of 1 × 10^–6^ M with respect to the initial amount. Reaction conditions: 2-MeTHQ (0.015 mmol, 2.2 μL), **E** (0.075 mmol, 26 mg), o-DCB (2 mL), 130 °C oil-bath temperature.

Upon incubation at 30 °C, together with the previous peaks, a new peak emerged at *m*/*z* 492.2 amu. This peak can be attributed to the formation of the epoxide intermediate named [Epo + H]^+^, arising from the attack of 2-MeTHQ to E (Figure S10). Upon increasing the temperature up to 130 °C, the [Epo + H]^+^ peak gradually disappeared, concomitant with the appearance of a new peak at *m*/*z* 144.0 amu, which was identified as the protonated form of the reaction product 2-MeQ, labeled [2-MeQ + H]^+^. These mass spectrometry data demonstrates that the acceptorless dehydrogenation of N-heterocycles proceeds through an initial epoxide intermediate, which is only observable at lower temperatures. No additional intermediates were detected during the course of the reaction, supporting a simplified two-step mechanism: the initial formation of an epoxide intermediate followed by its conversion to the dehydrogenated product under the given conditions.

### Detection of Intermediates by NMR Spectroscopy

To further investigate the reaction mechanism suggested by ESI-MS analysis, the stoichiometric reaction of THQ with *o*-quinone-type model molecules was studied using NMR spectroscopy. Specifically, the reaction of 1,2,3,4-tetrahydroisoquinoline (THiQ) with 9,10-phenanthrenequinone (G) was monitored by ^1^H NMR spectroscopy in MeCN-d3 using a high-pressure NMR tube (Figure 7). THiQ was chosen over tetrahydroquinolines for these experiments due to its slower reaction kinetics and distinct NMR features, which allow for the selective detection of the monodehydrogenated product, 3,4-dihydroisoquinoline (DHiQ).

**Figure 7:**
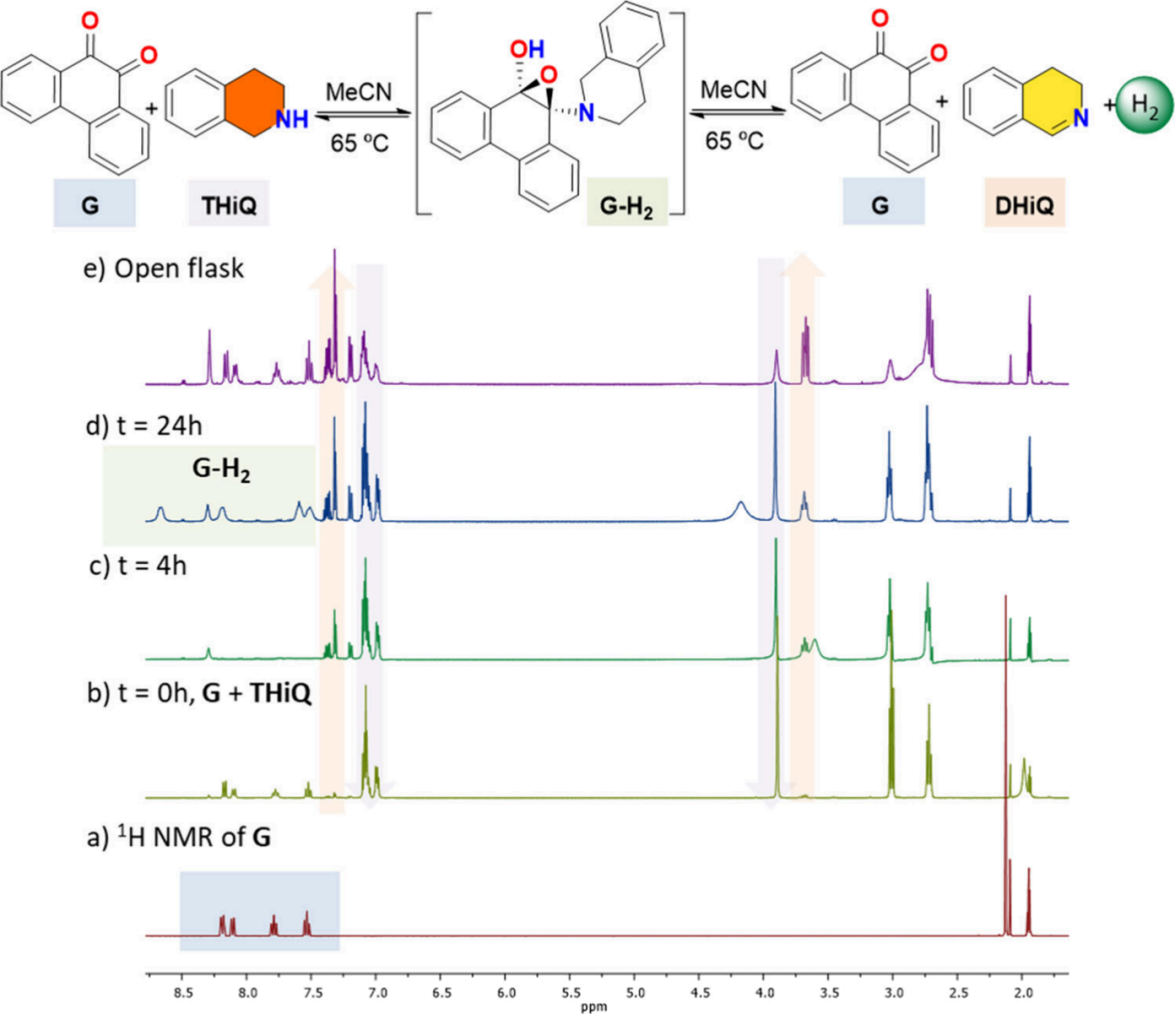
^1^H NMR spectroscopy monitoring of THiQ dehydrogenation using stoichiometric amounts of 9,10-phenanthrenequinone (G) in MeCN-d3. (a) ^1^H NMR spectrum of G used for comparison purposes, (b) *t* = 0 h reaction of G + THiQ highlighting the characteristic signals of THiQ and DHiQ, (c) *t* = 4 h reaction showing the initial formation of DHiQ, (d) reaction at *t* = 24 h, indicating the presence of G-H_2_ and (e) evolution of G-H_2_ into G upon opening the tube, with the concomitant formation of H_2_. Reaction conditions: THiQ (0.072 mmol, 8.2 μL), **G** (0.024 mmol, 5 mg), MeCN-d3 (0.75 mL), 65 °C oil-bath temperature.

At the initial stage of the reaction (t = 0 h), well-defined NMR peaks for both THiQ and 9,10-phenanthrenequinone (G) were observed. After 4 h at 65 °C, signals corresponding to the product DHiQ began to emerge (Figure 7c), while the peaks for G became significantly broadened and less intense. This broadening is attributed to the formation of a reversible epoxide intermediate, as previously inferred from ESI-MS data. Similar dynamic equilibria involving quinone intermediates have been reported by Stahl and co-workers in related systems involving 1,10-phenanthroline-5,6-dione and tetrahydroquinoline.^50,51^

Over the course of the 24-h reaction, a gradual decrease in THiQ signals and a corresponding increase in DHiQ signals were observed, indicating a conversion of approximately 40%. Notably, a new set of resonances emerged at this stage (Figure 7d), consistent with the formation of the epoxide intermediate (G-H_2_). These signals disappeared upon depressurization of the NMR tube, suggesting that hydrogen release drives the equilibrium toward product formation. Upon hydrogen release, the signals of 9,10-phenanthrenequinone (G) signals were restored, as confirmed by the ^1^H NMR pattern (Figure 7e). Chromatographic analysis (Figure S11) of the gas evolved during the reaction further corroborated the release of hydrogen from the epoxide intermediate (G-H_2_).

These results provide strong experimental support for a reversible epoxide intermediate as a key mechanistic step in the acceptorless dehydrogenation of N-heterocycles. The observed reversibility and hydrogen evolution underscore the central role of *o*-quinone functional groups as the catalytically active sites in this process.

Based on the reaction intermediates detected by NMR spectroscopy and ESI/MS studies, along with the use of quinone model molecules and masking experiments, a plausible reaction mechanism is proposed for the acceptorless dehydrogenation of N-heterocycles using graphene-like materials (Figure 8). These experimental results provide strong evidence that *o*-quinone groups function as active sites in dehydrogenation processes catalyzed by graphene-based materials.

**Figure 8:**
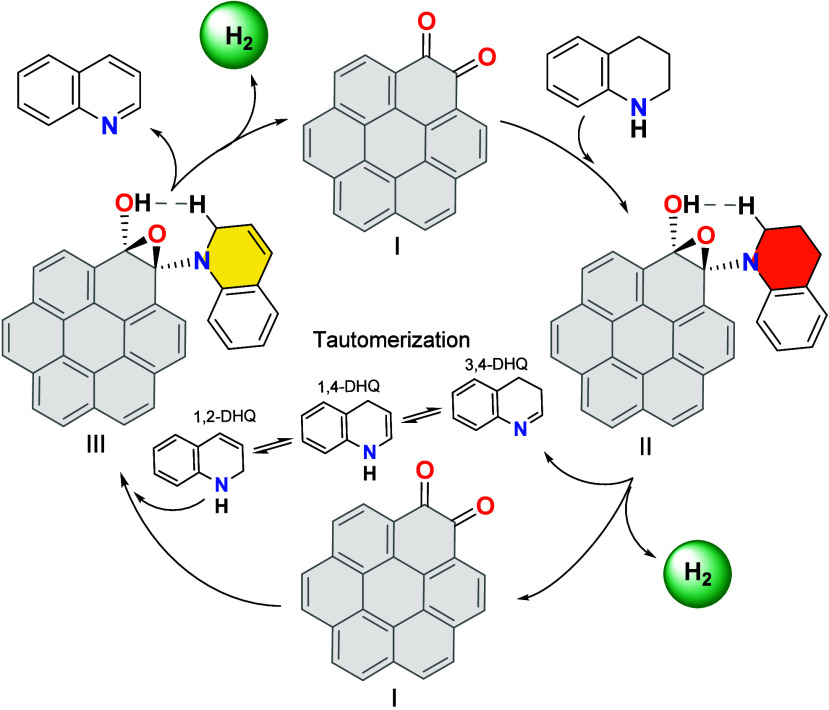
Mechanistic proposal for the acceptorless dehydrogenation of THQ using GNPs as carbocatalyst. The GNPs carbocatalyst is represented as a coronene-1,2-dione cluster model.

The dehydrogenation of tetrahydroquinolines begins with a nucleophilic attack by the NH group of the substrate on one of the carbonyls of the *o*-quinone, while the N–H proton is transferred to the adjacent carbonyl oxygen in a concerted step, resulting in the formation of an epoxide intermediate (II). The involvement of *o*-quinone functional groups in this step is corroborated by model compound studies (Figure 5), while the existence of intermediate II has been directly confirmed by ESI-MS analysis (Figure 6).

Subsequently, a key proton transfer occurs: the alcohol proton in intermediate II interacts with the hydrogen at the C2 position of the THQ backbone, leading to the formation of 3,4-dihydroquinoline (3,4-DHQ) and the release of molecular hydrogen. This step also restores the *o*-quinone group to its original state (II → I), thereby completing the catalytic cycle. The necessity of the C2-hydrogen in this transformation was previously demonstrated by our group using a quinoline derivative lacking a C2 proton, which showed no activity.^39^ Theoretical analysis further supports the feasibility of this hydrogen release mechanism (Figure 9).

**Figure 9:**
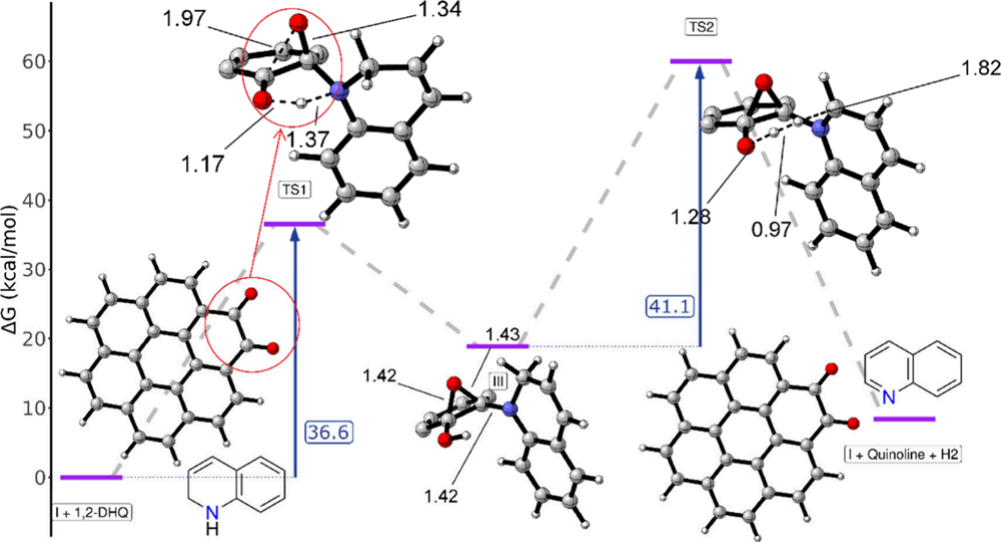
Potential Energy Surface (PES) for the dehydrogenation mechanism of 1,2-DHQ through the epoxide intermediate, computed at the PBE0/def2-tzvp level of theory with D3 dispersion and SMD solvation (o-DCB). Reaction steps correspond to the intermediates shown in Figure 8. For clarity, only key atoms are depicted in TS1, III and TS2. Reaction barrier values (TS1 and TS2) are shown explicitly, distances are given in Å. Color code: gray = carbon atoms; white = hydrogen atoms; red = oxygen atoms; blue = nitrogen atoms.

Following the initial dehydrogenation, tautomerization of 3,4-DHQ yields intermediate isomers such as 1,4- and 1,2-dihydroquinoline. The second dehydrogenation step mirrors the first: hydrogen is again released via a similar concerted mechanism involving the alcohol proton and the C2-hydrogen (I → III → I), ultimately affording fully aromatized quinoline and a second equivalent of hydrogen gas.

Together, these findings establish a mechanistic framework in which *o*-quinone groups embedded in the graphitic carbon network serve as redox-active sites, enabling reversible hydrogen transfer and driving the acceptorless dehydrogenation of N-heterocycles. This mechanistic insight not only aligns with all observed experimental data but also provides a valuable foundation for the rational design of future carbocatalysts based on graphene-like materials.

### Computational Results

To complement the experimental results, density functional theory (DFT) calculations were carried out to explore the mechanistic pathway of acceptorless dehydrogenation. The computational approach involved the modeling of key intermediates and transition states at the PBE0/def2-tzvp level of theory. Solvent effects were subsequently incorporated using the SMD model.

Experiments have been performed on a series of tetrahydroquinolines (2-methyltetrahydroquinoline, 2-MeTHQ, in Figure 1, several 1,2,3,4-tetrahydroquinolines, 1,2,3,4-THQ, in Figure 2, and 1,2,3,4-tetrahydroisoquinoline, THiQ in Figure 7) that provide dihydroquinolines (DHQ) as reaction intermediates. For the theoretical calculations, we have chosen 1,2-DHQ and modeled half of the reaction mechanism since the second half proceeds similarly. The half part of the reaction mechanism modeled corresponds to steps I → III → I (Figure 8).

The acceptorless dehydrogenation of THQ proceeds via two sequential dehydrogenation steps that follow a similar mechanistic route, the first step is the process from tetrahydroquinoline to 1,2-DHQ, and the second step corresponds to quinoline formation. As said above, only the second dehydrogenation step was modeled. To simplify the calculations, the α,β-dicarbonyl terminated graphene structure was represented by a coronene-1,2-dione cluster model (Figure 9). Further studies on smaller models such as pyrene-4,5-dione and phenantrene-9,10-dione clusters, as well as different density-functionals and solvent effects, were carried out for comparison and are detailed in the Supporting Information (Figure S12 and Table S5).

The energy profile begins with the interaction of coronene-1,2-dione with 1,2-DHQ, leading to the formation of an alcohol-epoxide intermediate (III) via the first transition state TS1 (Figure 9). Subsequently, interaction of the hydrogen atom from alcohol with H–C2 of 1,2-DHQ facilitates the release of molecular hydrogen, yielding quinoline and regenerating the catalyst coronene-1,2-dione. This step has the highest activation barrier due to the high energy required to cleave the C–H bond of the 2-position in 1,2-DHQ to yield H_2_ and quinoline. The calculated activation barrier for the rate-determining step is 41.1 kcal/mol (TS2) at the PBE0/def2-tzvp (with o-DCB SMD solvation) level of theory, which corresponds to the release of H_2_ and quinoline in a single, concerted step. This result is consistent with previous reports on acceptorless dehydrogenation of substrates such as ethylbenzene, with reported similar activation barriers using the same theoretical methods (Tables S6 and S7 offer a comparison between different DFT functionals of this reaction and reaction barriers from similar processes in the literature)^52,53^ Although the dehydrogenation of THQ is thermodynamically uphill (the computed reaction free energy change at 130 °C is +8.4 kcal/mol, Figure 9), it is entropically favored due to the release of hydrogen gas.

Other plausible reaction pathways (Section S13), that involve an initial transfer hydrogenation step were also evaluated, but they led to energy barriers largely exceeding those by the epoxide mechanism. Several DFT studies have reported dehydrogenation energies of butane to 1-butene using graphenes and nanodiamonds^54,55^ using the Generalized Gradient Aproximation (GGA) PBE functional.^56^ Although more computationally expensive, hybrid functionals, such as PBE0,^57^ used in this work, often yield more accurate energy values.^58^ For comparison, the energies at the PBE/def2-tzvp level of theory, with D3 dispersion correction, were also evaluated, as well as with other DFT functionals (Tables S6 and S7).

## Conclusions

We have identified that o-quinone moieties in graphene-based carbocatalysts can promote the dehydrogenation of N-heterocycles, accompanied by the release of molecular hydrogen. These o-quinone groups facilitate both dehydrogenation and hydrogen release through the formation of epoxide intermediates. Using model molecules with various quinone functionalities, along with masking experiments and reaction monitoring of stoichiometric interactions between substrate and organocatalyst, direct spectroscopic and spectrometric evidence supporting the intermediacy of epoxide species was obtained. The experimental results related to epoxide formation are further supported by theoretical studies using DFT calculations. The acceptorless dehydrogenation of N-heterocycles presents a sustainable alternative to conventional oxidation methods and holds potential for hydrogen storage via the formation of covalent bonds.

## Experimental Section

### Materials

N-heterocycles and GNPs were purchased from commercial suppliers and used without further purification. Anhydrous solvents were dried using a solvent purification system or purchased from commercial suppliers and stored over molecular sieves. Solvents were deoxygenated using the freeze–pump–thaw methodology and kept under an atmosphere of nitrogen. Instrumentation, characterization and detailed experimental procedures are included in the Supporting Information.

### General Procedure for Catalytic Dehydrogenation Reactions

All catalytic experiments were carried out under an inert atmosphere of nitrogen using dry and deoxygenated solvents. In a typical procedure, the carbocatalyst (graphenes) or organocatalyst (model molecules) was filled into a dry Schlenk flask (20 mL). The reaction system is connected to a bubbler filled with mineral oil to exclude air and allow the release of hydrogen gas. Solvent (1 mL), substrate (0.15 mmol) and 1,3,5-trimethoxybenzene as a standard were added under a nitrogen flux. The mixture was stirred at 130 °C (bath temperature) for at least 24 h. The reaction progress was monitored by gas chromatography taking aliquots at selected intervals measuring the liquid phase.

### Theoretical Studies

Computational calculations were performed using Gaussian 09 Rev D.01.^59^ All geometry optimizations were performed without any symmetry constraints, by means of the Berny algorithm.^60^ Energy minima were confirmed through frequency analysis showing no imaginary frequency, while Transition State (TS) structures were confirmed with exactly one imaginary frequency. The connectivity between stationary points was established by intrinsic reaction path calculations (IRC). Calculations were carried out at diverse levels of theory (PBE,^56^ PBE0,^57^ M06-2X,^61^ and wB97XD^62^) with def2-tzvp^63^ basis sets and Grimme’s D3 dispersion corrections^64^ (if no dispersion correction is already present in the functional). Solvent effects were included with the SMD^65^ model to mimic the solvation effects of o-DCB during both geometry optimizations and vibrational analysis. Free energy values were computed specifying a temperature of 130 °C to better compare with the experimental values. Results in the main manuscript are those from PBE0/def2-tzvp with SMD solvation.

## Supplementary Material



## Notes

Additional characterization of GNPs by XPS, Raman, TGA and HRTEM; further details of mechanistic studies (experimental procedures, NMR characterization, ESI-MS and trapping experiments), and details of DFT calculations (PDF)
