# Supplementary material for: Mechanistic Insights in Acceptorless Dehydrogenation of N‑Heterocycles Using Graphenes as Carbocatalysts
Source: ACS Appl Mater Interfaces. 2025 Dec 13;17(51):69234–44. doi: 10.1021/acsami.5c11895 (PMC12754750; doi:10.1021/acsami.5c11895)
Supplement: Supplementary file 1 [file am5c11895_si_001.pdf]

SUPPORTING INFORMATION

Mechanistic Insights in Acceptorless Dehydrogenation of N-Heterocycles Using Graphenes as Carbocatalysts

Andrés Mollar-Cuni,[a] Pablo García-Aznar,[b] Santiago Martín,[c,d,e] German Sastre[b] Hermenegildo García[b] and Jose A. Mata*[a]*

[a]Institute of Advanced Materials (INAM), Universitat Jaume I, Avda. Sos Baynat s/n, 12006, Castellón (Spain). Tel: +34 964387516. Email: jmata@uji.es

[b]Instituto de Tecnología Química, Consejo Superior de Investigaciones Científicas-Universitat Politècnica de València. Avda. Los Naranjos s/n, 46022, Valencia (Spain). Email: hgarcia@qim.upv.es

[c]Instituto de Nanociencia y Materiales de Aragón (INMA), CSIC-Universidad de Zaragoza, 50009, Zaragoza (Spain)

[d]Departamento de Química Física, Universidad de Zaragoza, 50009, Zaragoza (Spain).

[e]Laboratorio de Microscopias Avanzadas (LMA). Universidad de Zaragoza, Edificio I+D+i. 50018, Zaragoza (Spain)

Contents

S1. General procedures 2
S2. Detection of molecular hydrogen 3
S3. X-ray photoelectron spectroscopy (XPS) 4
S4. Raman spectroscopy 5
S5. Thermogravimetric analysis of GNPs 6
S6. Microscopic characterization of GNPs 7
S7. Correlation of carbonyl groups (C=O) in GNPs vs. activity 8
S8. Masking experiments 9
S8.1 Synthesis and characterization of dibenzo[f,h]quinoxaline 9
S8.2 Synthesis and characterization of $GNP750_{NEtN}$ 10
S9. Experimental procedure for epoxide detection by ESI/MS 11
S10. Experimental procedure for epoxide detection by NMR spectroscopy. 12
S11. ^{1}H NMR spectra of organic products 13
S12. Effect of cluster size. Epoxide mechanism evaluation. 15
S13. DFT reaction mechanisms 16
S14. DFT density functional comparison. 20
S15. Characterization of model molecules used as organocatalyst. 22
S16. References 26

S1. General procedures

Reagents and solvents. N-heterocycles were purchased from commercial suppliers and used without further purification. GNPs were obtained from XG Science, Inc. (Grand Oak Drive, Lansing, MI, USA). Anhydrous solvents were dried using a solvent purification system or purchased from commercial suppliers and stored over molecular sieves. Solvents were deoxygenated using the freeze-pump-thaw methodology and kept under an atmosphere of nitrogen.

Instrumentation. Nuclear magnetic resonance (NMR) spectra were recorded on Bruker spectrometers operating at 400 MHz (^{1}H NMR) and 100 MHz ($^{13}C\{^{1}H\}$ NMR), respectively, and referenced to $SiMe_4$ (δ in ppm and J in Hertz). NMR spectra were recorded at room temperature with the appropriate deuterated solvent. Elemental Analysis was carried out in a TruSpec Micro Series. High-resolution images of transmission electron microscopy (HRTEM) and high-angle annular dark-field (HAADF-STEM) images of the samples were obtained using a Jem-2100 LaB6 (JEOL) transmission electron microscope coupled with an INCA Energy TEM 200 (Oxford) energy dispersive X-Ray spectrometer (EDX) operating at 200 kV. Samples were prepared by drying a droplet of a MeOH dispersion on a carboncoated copper grid. X-ray photoelectron spectra (XPS) were acquired on a Kratos AXIS ultra DLD spectrometer with a monochromatic Al Kα X-ray source (1486.6 eV) using a pass energy of 20 eV. To provide a precise energy calibration, the XPS binding energies were referenced to the C1s peak at 284.6 eV. Gas chromatography (GC) analyses were obtained on a shimadzu GC-2010 apparatus equipped with a FID detector, and using a Teknokroma column (TRB-5MS, 30 m x 0.25 mm x 0.25 µm). Thermogravimetric analyses (TGA) were performed using a TG-STDA Mettler Toledo model TGA/SDTA851e/LF/1600 coupled to a mass spectrometer quadrupol PFEIFFER VACUUM model OmniStar GSD 320 O3, 1-300 uma bearing a tungsten filament.

S2. Detection of molecular hydrogen

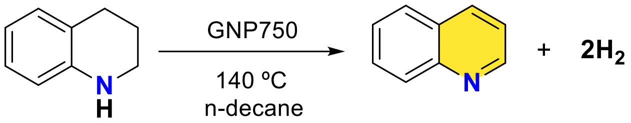

Experimental conditions: Hydrogen could not be detected under the standard reaction conditions, most likely due to its high dilution. To facilitate detection, the reaction scale was increased: 1,2,3,4-tetrahydroquinoline (1 mmol, 125.5 µL), n-decane (6 mL) and GNP750 (120 mg). The reagents were placed in a Schlenk flask (50 mL) equipped with a reflux condenser connected via a trap flask (250 mL Schlenk flask) to an oil bubbler to prevent the entrance of oxygen and allowing the release of hydrogen. The system was purged with argon and immersed in an oil bath preheated to 140 °C. After 2h reaction, the gases collected in the trap flask were analysed by gas chromatography with a thermal conductivity detector (GC-TCD) (Agilent 990 Micro-GC gas chromatograph) using argon as carrier gas. The GC-TCD spectrum confirmed the formation of molecular hydrogen from the reaction.

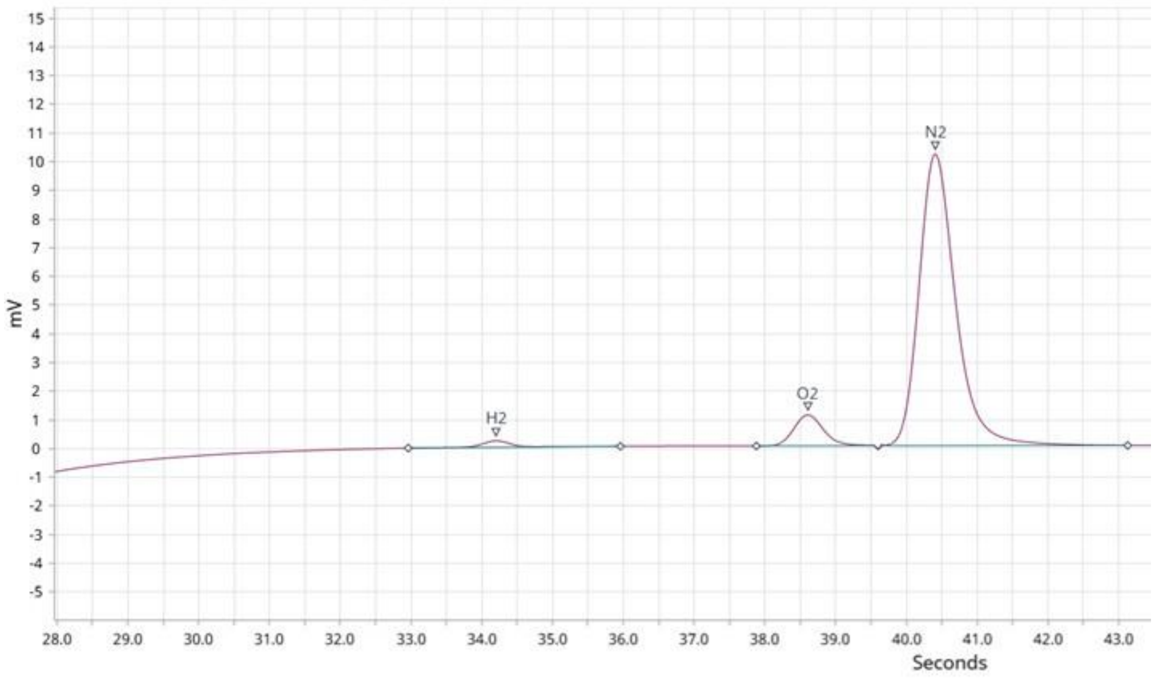

Figure S1 GC-TCD spectrum of the gas phase showing the presence of H_2 (O_2 and N_2 are from the measurement process) after 2h reaction in dehydrogenation of 1,2,3,4-tetrahydroquinoline.

S3. X-ray photoelectron spectroscopy (XPS)

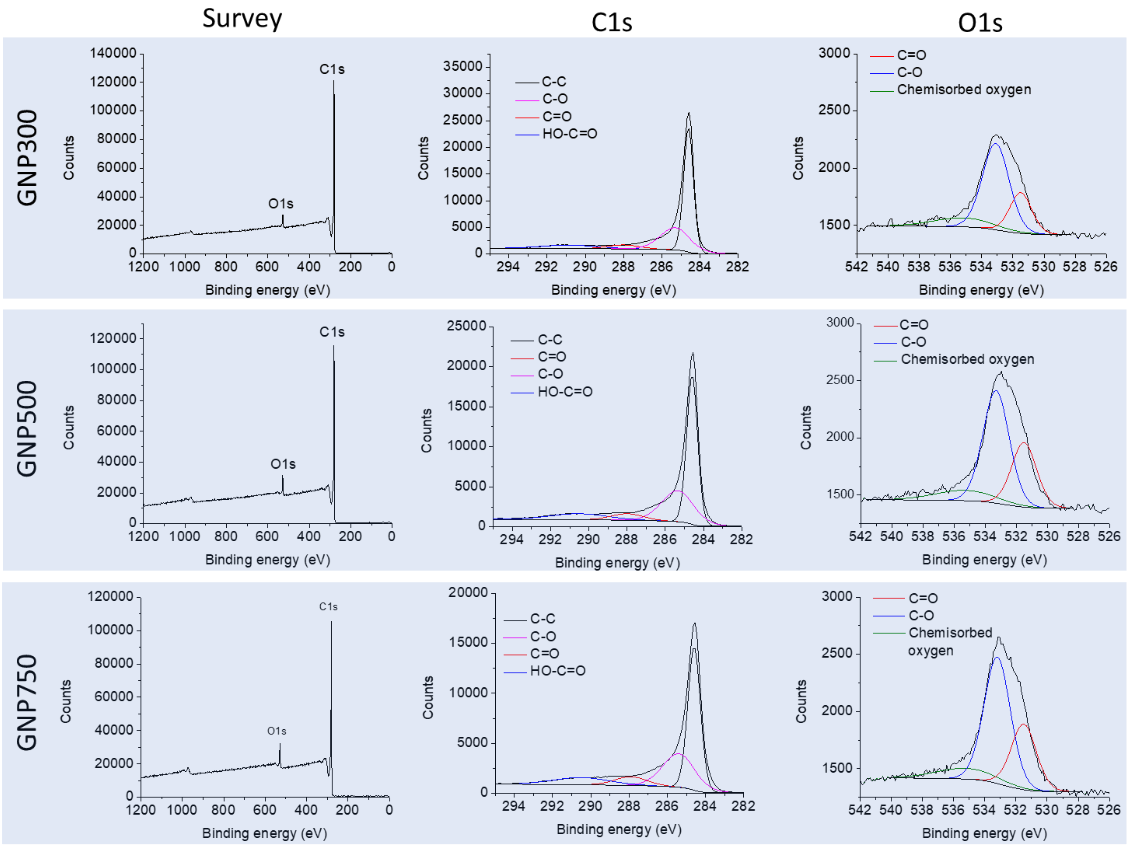

Figure S2 XPS analysis of GNPs (300, 500 and 750) showing the survey spectrum and the high-resolution peak of C1s and O1s.

S4. Raman spectroscopy

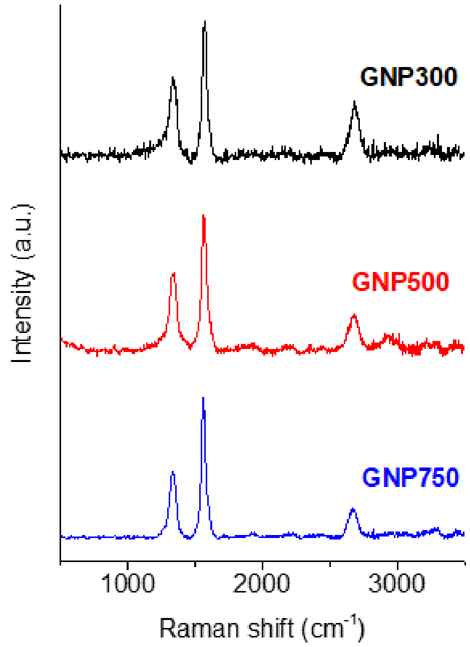

Figure S3 Raman spectra of GNP300, GNP500 and GNP750 showing the predominant D (1350 cm^{-1}), G (1560 cm^{-1}) and 2D (2700 cm^{-1}) bands.

Table S1 Intensity ratio between graphitic (G) and defective (D) bands.

Sample	I_G/I_D
GNP300	1.6
GNP500	1.7
GNP750	2.1
GNP750$_{NEtN}$	5.0

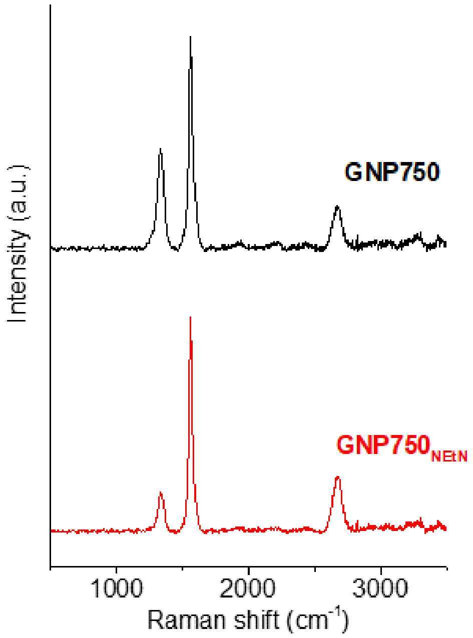

Figure S4 Comparative Raman spectra of GNP750 and GNP750$_{NEtN}$.

S5. Thermogravimetric analysis of GNPs

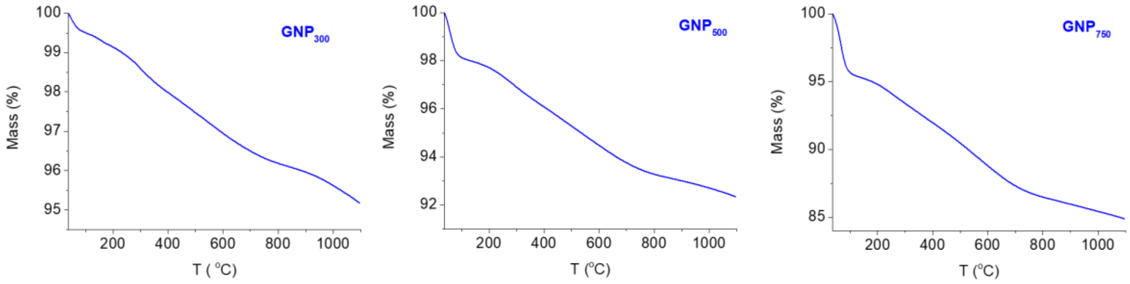

Figure S5 Thermogravimetric analysis of GNPs under an inert N_2 atmosphere.

Table S2 Analysis of mass losses at different temperatures.

Sample	Water losses (Tª < 150 °C) (wt %)	Mass losses (wt %)	Total losses (wt %)
**GNP300**	0.7	4.1	4.8
**GNP500**	2.1	5.6	7.7
**GNP750**	4.8	10.3	15.1

Table S3 Elemental analysis (wt %) of GNP materials after TGA analysis.

Sample	C	N	H	O	Ratio O/C
**GNP300**	97.9	0.2	0.3	1.6	0.02
**GNP500**	97.9	0.3	0.3	1.5	0.02
**GNP750**	96.0	0.5	0.3	3.2	0.03

S6. Microscopic characterization of GNPs

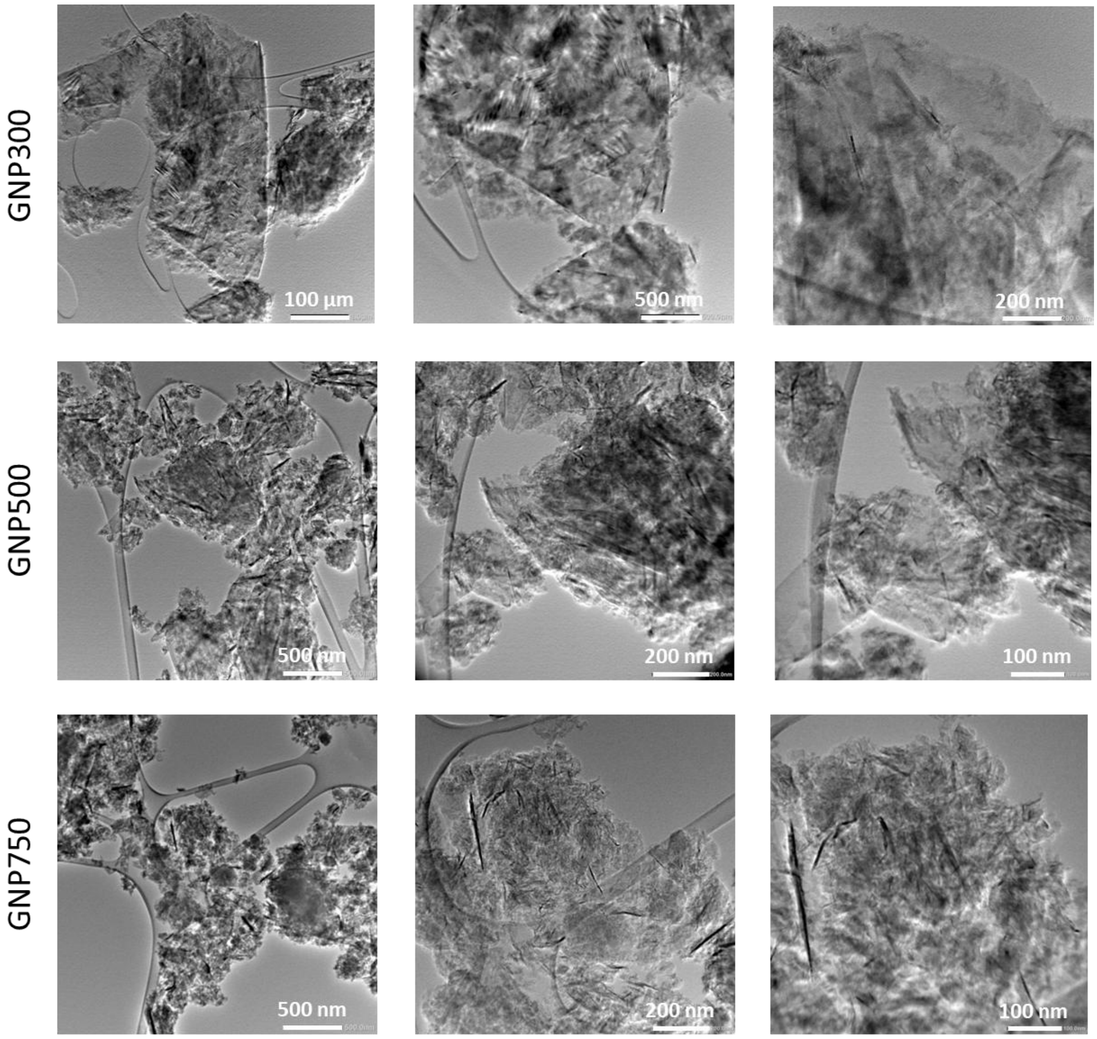

Figure S6 HRTEM images of GNPs at different magnifications.

S7. Correlation of carbonyl groups (C=O) in GNPs vs. activity

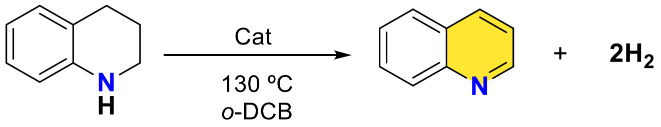

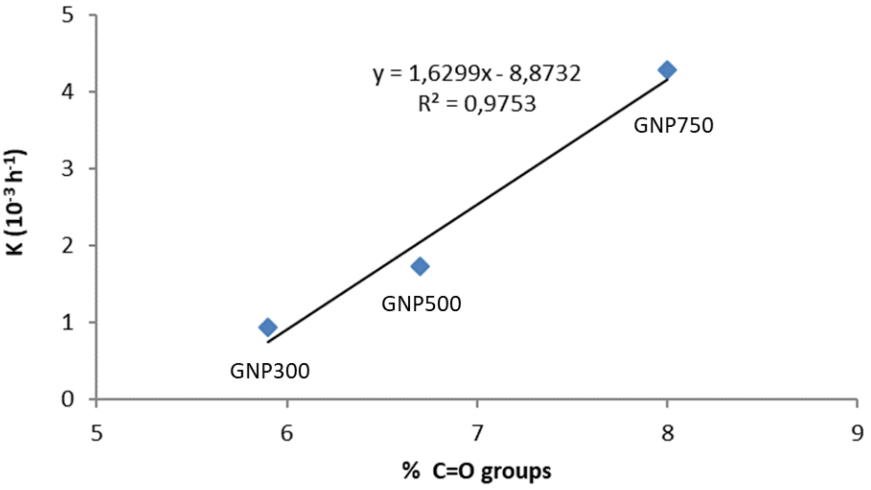

Figure S7 Linear correlation between reaction rate constants (Figure 1a) and %C=O groups (Figure 1c) of GNP750, GNP500 and GNP300.

S8. Masking experiments

S8.1 Synthesis and characterization of dibenzo[f,h]quinoxaline

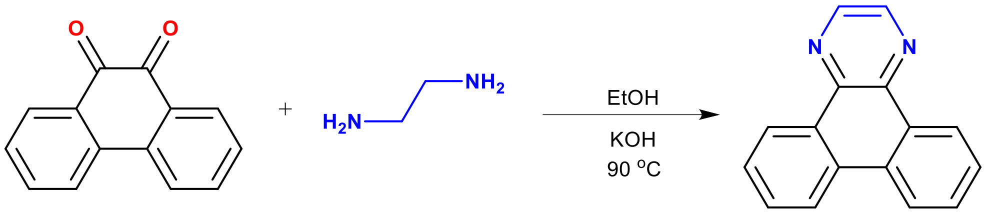

The dibenzo[f,h]quinoxaline was synthesized by adapting a previous reported procedure in the literature.[1] 9,10-Phenanthrenequinone (150 mg, 0.075 mmol) and KOH (45 mg, 0.8 mmol) were filled into a dry high pressure Schlenk flask (100 mL) tube and deoxygenated through vacuum/N_2 cycles. The solids were dissolved in 20 mL of dry ethanol. Then, ethylenediamine (56.6 µL, 0.8 mmol) was added under N_2 flux. The mixture was stirred under reflux for 18h. Then, the solvent was removed under reduced pressure. The residue was dissolved in dichloromethane and washed with water (x3). The organic layers were dried over $MgSO_4$ and concentrated under vacuum. The product was purified by silica gel column chromatography (dichloromethane) to afford 124 mg (74%) of dibenzo[f,h]quinoxaline as a yellow solid.

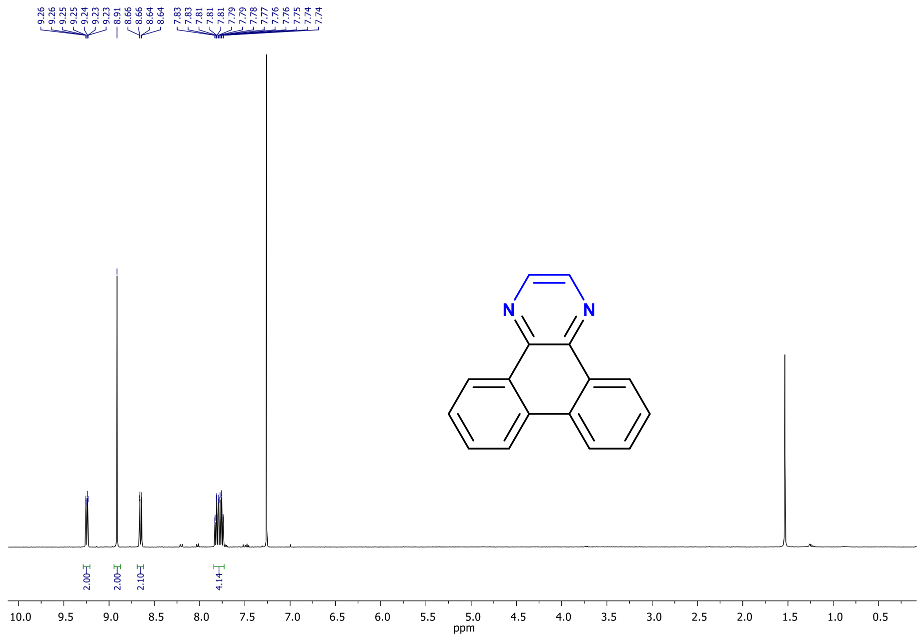

Figure S8 ^1H NMR spectrum of dibenzo[f,h]quinoxaline in $CDCl_3$ (7.26 ppm). Signal at 1.65 ppm corresponds to residual water.

S8.2 Synthesis and characterization of $GNP750_{NEtN}$

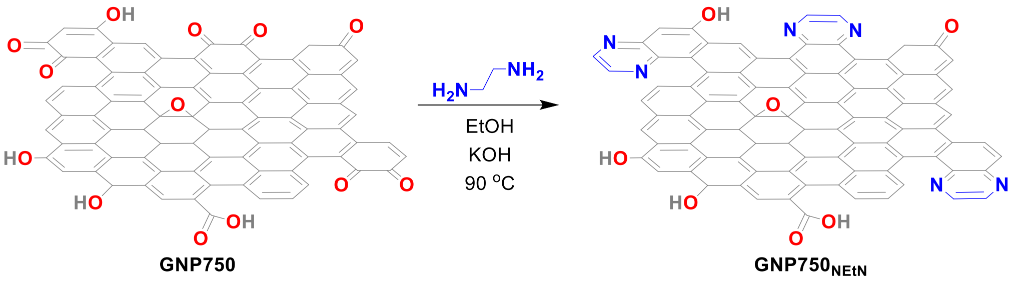

The material was synthesized following the methodology described in section S7.1 for the preparation of dibenzo[f,h]quinoxaline. In brief, 150 mg of GNP750, 60 mg of KOH and 20 mL of dry ethanol were introduced into a high pressure Schlenk under N_2. Then, 120 µL of ethylenediamine were added dropwise to the solution. The suspension was stirred at 90 °C during 16h. After this time, the suspension was filtered and washed with water, ethanol, ether and pentane providing a black powder.

Table S4 Elemental analysis (wt %) of fresh and used $GNP750_{NEtN}$

Sample	C	N	H	O	Ratio O/C
**$GNP750_{NEtN}$**	78.5	1.3	0.9	19.3	0.24
**$GNP750_{NEtN}$ 1run**	80.5	1.5	1.8	17.2	0.21

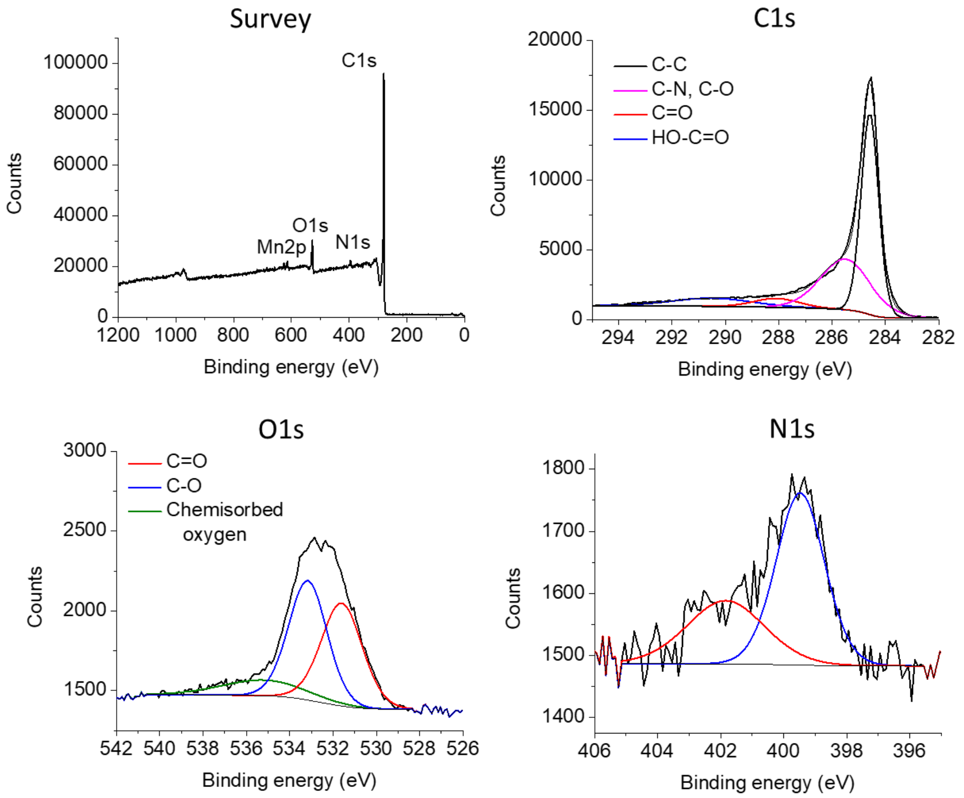

Figure S9 XPS analysis of $GNP750_{NetN}$ showing the survey spectra and high-resolution C 1s, O 1s and N 1s.

S9. Experimental procedure for epoxide detection by ESI/MS

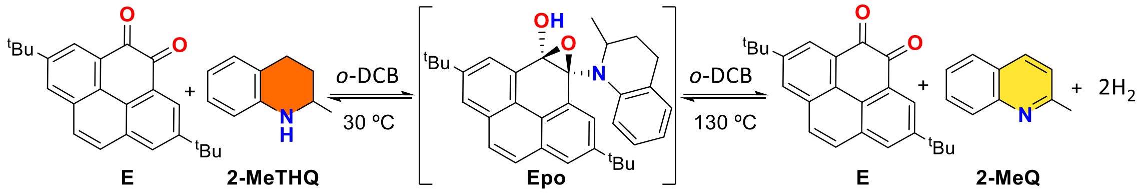

2,7-bis(tert-butyl)-4,5-pyrenedione (**E**) (26 mg, 0.075 mmol) was filled into a dry Schlenk flask (20 mL). Solvent (o-dichlorobenzene, 2 mL) and 2-Methyl-1,2,3,4-tetrahydroquinoline (2-MeTHQ) (2.2 µL, 0.015 mmol) were added under a nitrogen flux. The mixture was stirred at 30 °C (bath temperature) for 24h. After this time, an aliquot was taken under N_2 and analyzed by mass spectrometry. Then, the reaction was heated at 130 °C (bath temperature) and connected to a condenser containing a bubbler filled with mineral oil. The bubbler excludes air from the reaction system while allowing the release of hydrogen gas displacing the equilibrium towards product formation. The reaction progress was monitored by mass spectrometry taking aliquots at different time intervals.

Peak (m/z)	Experimental mass	Theoretical mass	Relative error (ppm)
492.29	492.2981	492.2903	15
492.29	493.2979	493.2936	9

Relative error (ppm) = [(theo – exp)/theo]*10^6

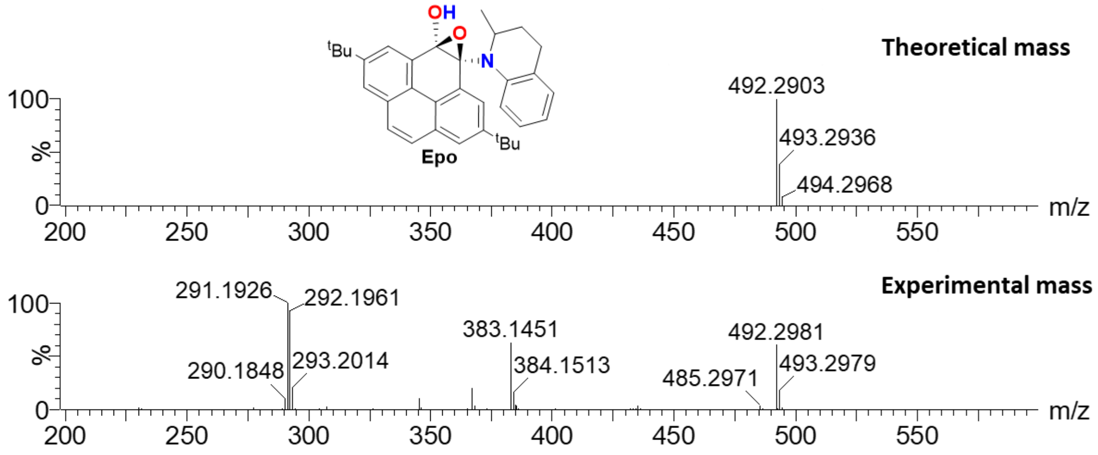

Figure S10 Experimental ESI-MS spectrum corresponding to the $[EPo + H]^+$ peak at *m/z* 492.29 together with the simulated isotopic pattern of this peak.

S10. Experimental procedure for epoxide detection by NMR spectroscopy.

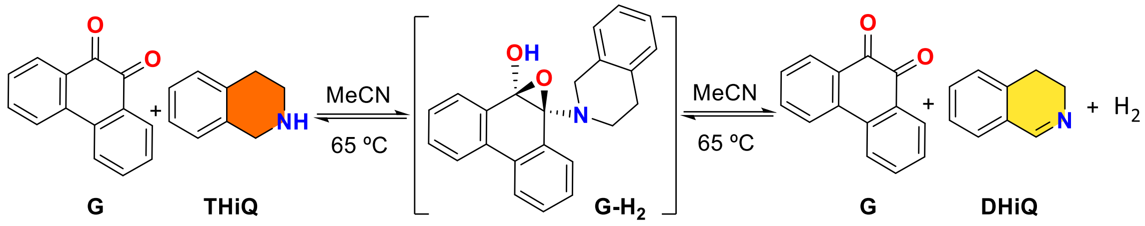

9,10-phenanthrenequinone *(***G)** (5 mg, 0.024 mmol) was introduced in an NMR tube and deoxygenated through vacuum/N_2 cycles. Dry and deoxygenated THiQ (8.2 µL, 0.072 mmol) and MeCN-d^3 (0.75 mL) were added under N_2. Then, the mixture was stirred at 65 ºC for 27 h. Reaction evolution was monitored by ^{1}H NMR spectroscopy at selected times.

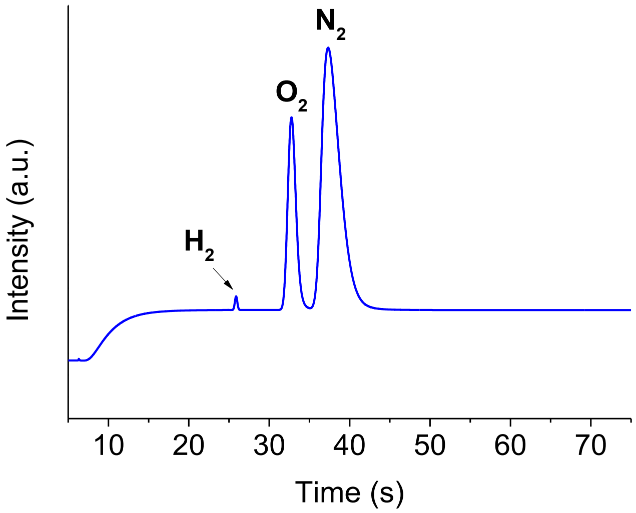

Figure S11 MicroGC spectrum after opening the NMR tube and heating at 65 °C showing the presence of H_2 (r.t. = 25 s).

S11. ^{1}H NMR spectra of organic products

Quinoline

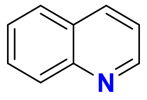

^{1}H NMR ($CDCl_3$, 400 MHz): δ 8.92 (dd, 1H, J = 4.2, 1.6 Hz), 8.15 (dd, 1H, J = 8.8, 1.3 Hz), 8.13 (d, 1H, J = 8.6 Hz), 7.82 (dd, 1H, J = 8.2, 0.8 Hz), 7.72 (ddd, 1H, J = 8.4, 6.9, 1.4 Hz), 7.55 (ddd, 1H, J = 8.5, 7.2, 1.1 Hz), 7.39 (dd, 1H, J= 8.2, 4.2 Hz).

6-Methylquinoline

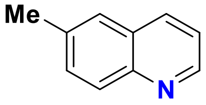

^{1}H NMR ($CDCl_3$, 400 MHz): δ 8.85 (d, 1H, 1.0 Hz), 8.07 (dd, 1H, J = 8.3, 0.9 Hz), 8.00 (d, 1H, J = 8.5 Hz), 7.61-7.51 (m, 2H), 7.36 (dd, 1H, J= 8.3, 4.2Hz), 2.54 (s, 3H).

6-Methoxyquinoline

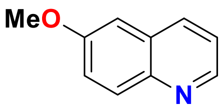

^{1}H NMR ($CDCl_3$, 400 MHz): δ 8.75 (dd, 1H, J = 4.3, 1.7 Hz), 8.03 (dd, 1H, J = 8.4, 1.1 Hz), 8.00 (d, 1H, J = 9.3 Hz), 7.38-7.31 (m, 2H), 7.05 (d, 1H, J= 2.8Hz), 3.91 (s, 3H).

6-Chloroquinoline

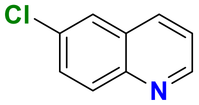

^{1}H NMR ($CDCl_3$, 400 MHz): δ 8.88 (dd, 1H, J = 4.2, 1.8 Hz), 8.06-8.03 (m, 2H), 7.81 (s, 1H), 7.65-7.63 (m, 1H), 7.42-7.39 (m, 1H).

7-Nitroquinoline

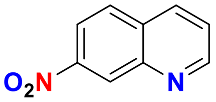

^{1}H NMR ($CDCl_3$, 400 MHz): δ 9.09 (dd, 1H, J = 3.9, 1.5 Hz), 9.01 (d, 1H, J = 1.6 Hz), 8.33 (dd, 1H, J = 8.9, 2.1 Hz), 8.28 (d, 1H, J = 8.3 Hz), 7.98 (d, 1H, J = 8.9 Hz), 7.60 (dd, 1H, J = 8.3, 3.4 Hz).

7-(Trifluoromethyl)quinoline

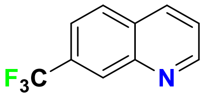

^{1}H NMR ($CDCl_3$, 400 MHz): δ 8.99 (dd, 1H, J = 4.2, 1.7 Hz), 8.39 (d, 1H, J = 0.5 Hz), 8.19 (dd, 1H, J = 8.4, 0.9 Hz), 7.91 (d, 1H, J = 8.6 Hz), 7.69 (dd, 1H, J = 8.5, 1.8 Hz), 7.49 (q, 1H, J = 4.2 Hz).

Dihydroisoquinoline

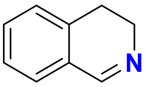

^{1}H NMR ($CDCl_3$, 400 MHz): δ 8.34 (s, 1H), 7.37-7.27 (m, 3H), 7.16 (d, 1H, J = 7.4 Hz), 3.78 (td, 2H, J = 8.0, 2.2 Hz), 2.76 (t, 2H, J = 8.0Hz),

Isoquinoline

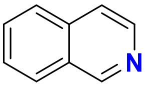

^{1}H NMR ($CDCl_3$, 400 MHz): δ 9.23 (s, 1H), 8.50 (d, 1H, J = 5.8), 7.93 (d, 1H, J = 8.2 Hz), 7.78 (d, 1H, J = 8.2 Hz), 7.69-7.63 (m, 1H), 7.61 (d, 1H, J = 5.8), 7.57 (ddd, 1H, J= 8.1, 6.9, 1.1 Hz).

Indole

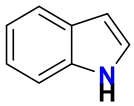

<u>^{1}H NMR ($CDCl_3$, 400 MHz)</u>: δ 8.14 (s, 1H), 7.67 (d, 1H, J = 7.8), 7.41 (d, 1H, J = 8.0 Hz), 7.24-7.18 (m, 2H), 7.14 (t, 1H, J = 7.4 Hz), 6.58 (brs, 1H).

9-*H*-Carbazol

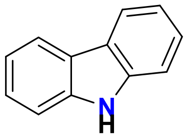

<u>^{1}H NMR ($CDCl_3$, 400 MHz)</u>: δ 8.09 (d, 2H, J = 8.30 Hz), 8.04 (brs, 1H), 7.45-7.41 (m, 4H), 7.26-7.23 (m, 2H).

β–Carboline

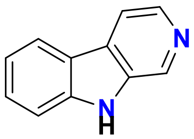

<u>^{1}H NMR (DMSO, 400 MHz)</u>: δ 11.62 (brs, 1H), 8.49 (dd, 1H, J = 7.8, 1.6 Hz), 8.41 (d, 1H, J = 4.8, 1.6 Hz), 8.15 (d, 1H, J = 7.8 Hz), 7.50 (d, 1H, J = 7.8 Hz), 7.45 (td, 1H, J = 7.8, 1.6 Hz), 7.25-7.17 (m, 2H).

S12. Effect of cluster size. Epoxide mechanism evaluation.

To better evaluate the effect of the graphene cluster size on the reaction mechanism, the reaction path (epoxide intermediate mechanism) was compared with a wide set of DFT functionals (M06-2X,[2] wB97XD,[3] PBE,[4] and PBE0[5]) and SMD solvation model[6] with o-DCB (Table S5). To assess the suitability of the small phenanthrene model as a graphene analogue, we also examined larger graphene models—pyrene-4,5-dione and coronene-1,2-dione—to evaluate potential energy variations arising from the extended π-conjugation present in bulk graphene. All three organocatalysts are shown in Figure S12. As Table S5 shows, very similar barriers are found regardless of the size of the organocatalyst employed in the calculations, which indicates that all models can be used for an accurate description of the reaction mechanism.

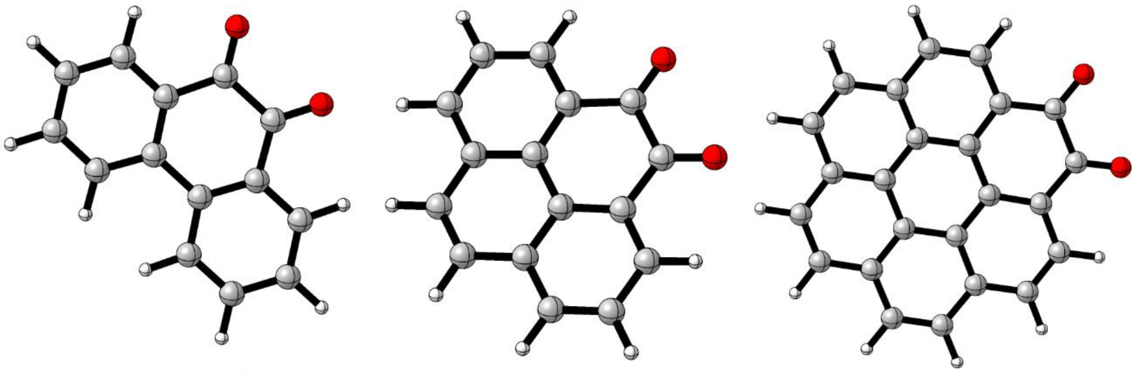

Figure S12 Structures of the different graphene cluster models employed in this study. Colo code: grey = carbon atoms; white = hydrogen atoms; red = oxygen atoms.

Table S5 Activation free-energy barriers (kcal/mol) of the epoxide mechanism (section S13.3, Figure S15) for different cluster models (Figure S12) and functionals.

		Epoxide mechanism		
Functional	Step	Phenantrene	Pyrene	Coronene
M06-2x-D3	Barrier 1	42,1	42,4	44,1
	Barrier 2	52,3	52,2	52,0
M06-2x-D3 + SMD[a]	Barrier 1		39,4	41,0
	Barrier 2		45,9	46,0
wB97XD	Barrier 1	42,5	43,4	45,0
	Barrier 2	50,9	50,7	50,5
PBE-D3	Barrier 1	34,0	34,1	35,7
	Barrier 2	35,5	35,4	35,2
PBE-D3 + SMD	Barrier 1		34,4	36,6
	Barrier 2		32,7	32,5
PBE0-D3	Barrier 1	35,6	36,1	37,4
	Barrier 2	45,9	45,8	45,6
PBE0-D3 + SMD	Barrier 1		36,2	36,6
	Barrier 2		40,9	41,1

[a]Solvation Model based on Density, using *o*-DCB parameters[6]

S13. DFT reaction mechanisms

In order to determine the reaction mechanism of the process, we considered three plausible routes, with a smaller (than coronene-1,2-dione) graphene cluster made of 9,10-phenanthrenequinone (I) (with no solvation model), using three different DFT functionals: PBE0,[5] PBE,[4] and M06-2X,[2] all with D3 dispersion correction,[7] and def2-tzvp basis sets.[8] The plausible routes are as follows:

S13.1 First (unsuccessful) reaction mechanism: Diol mechanism

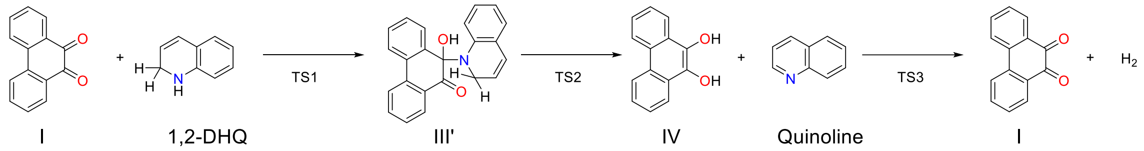

Scheme S1. Reaction mechanism involving the hemiaminal intermediate (III'), yielding a diol termination (IV) and quinoline.

The diol reaction mechanism involves the formation of a hemiaminal species (III') from 9,10-phenanthrenequinone and 1,2-DHQ. This hemiaminal would then protonate the adjacent carbonyl group, carrying out a full "transfer hydrogenation" step that yields a diol species and quinoline. The release of molecular hydrogen was evaluated from the diol intermediate. The calculated transition state (TS3) associated with hydrogen evolution from this intermediate was found to lie with an activation energy of 69.0 kcal/mol above the energy of intermediate IV, rendering the formation of quinone (I) and H_2 thermodynamically inaccessible under the reaction conditions. The resulting potential energy surface (PES) is shown in Figure S13.

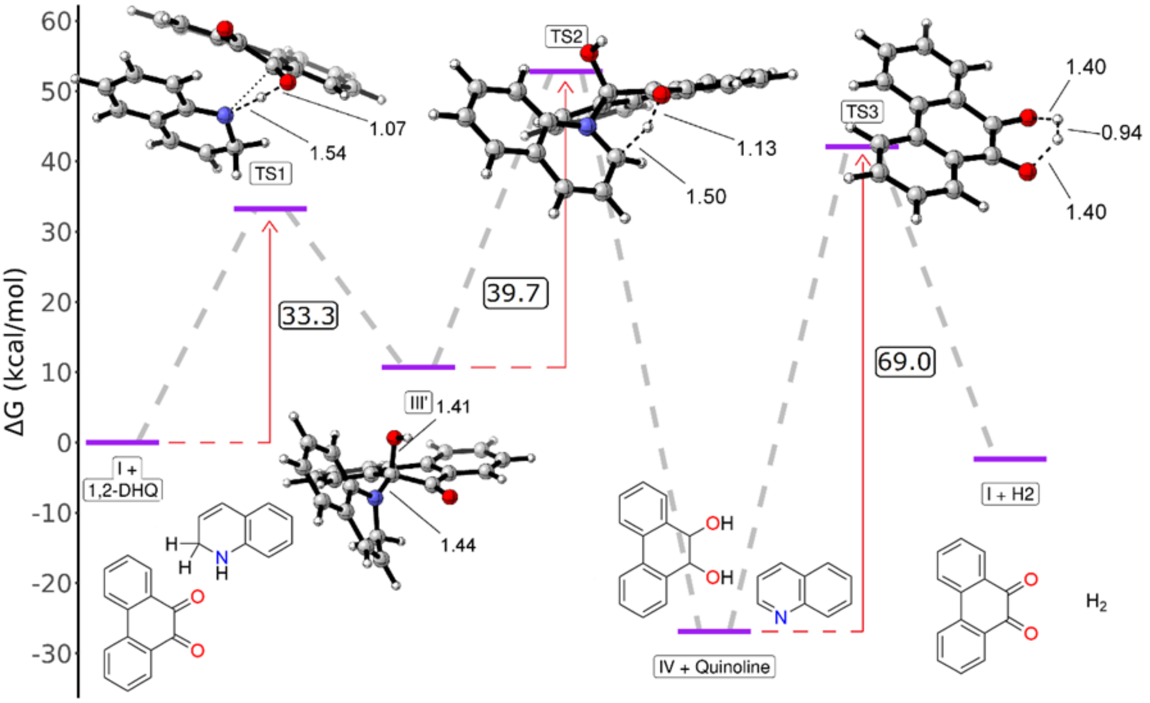

Figure S13 Potential Energy Surface (PES) of the diol mechanism using PBE0/def2-tzvp. Reaction intermediates labelled as in Scheme S1. Distances are given in Å. Color code: grey = carbon atoms; white = hydrogen atoms; red = ocygen atoms; blue = nitrogen atoms.[9,10]

S13.2 Second (unsuccessful) reaction mechanism: Hemiaminal mechanism

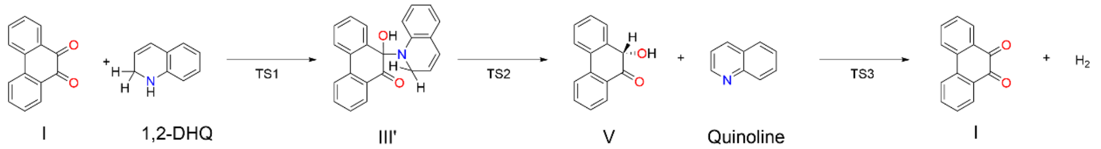

Scheme S2. Reaction mechanism involving the hemiaminal intermediate (III'), yielding a ketone with an alpha alcohol group (V) and quinoline.

This reaction mechanism is equivalent to the first reaction mechanism until step III'. From this hemiaminal intermediate (III'), the deprotonation of the 1,2-DHQ occurs at the carbonyl carbon of the contiguous ketone group, yielding quinoline. The protonated carbonyl group will readily pick the H from the contiguous OH group yielding 10-hydroxyphenantren-9(10H)-one (V) and regenerating the ketone group. The overall hydrogen transfer occurs from 1,2-DHQ to the graphene, yielding quinoline and the hydrogenated graphene species.

Although the two hydrogens (OH and H) bonded to the same carbon are relatively close, the formation of H_2 was not favorable as we observed in the first reaction mechanism. The transition state was found with an activation energy of 87.1 kcal/mol , way above any feasible reaction at the reaction temperatures, and being the highest overall barrier of all studied paths (Figure S14). This mechanism is analogous to that proposed by Feng *et. al.,* in which they observed hydrogen gas following this pathway using nanodiamond as carbocatalysts containing dione terminations.[11]

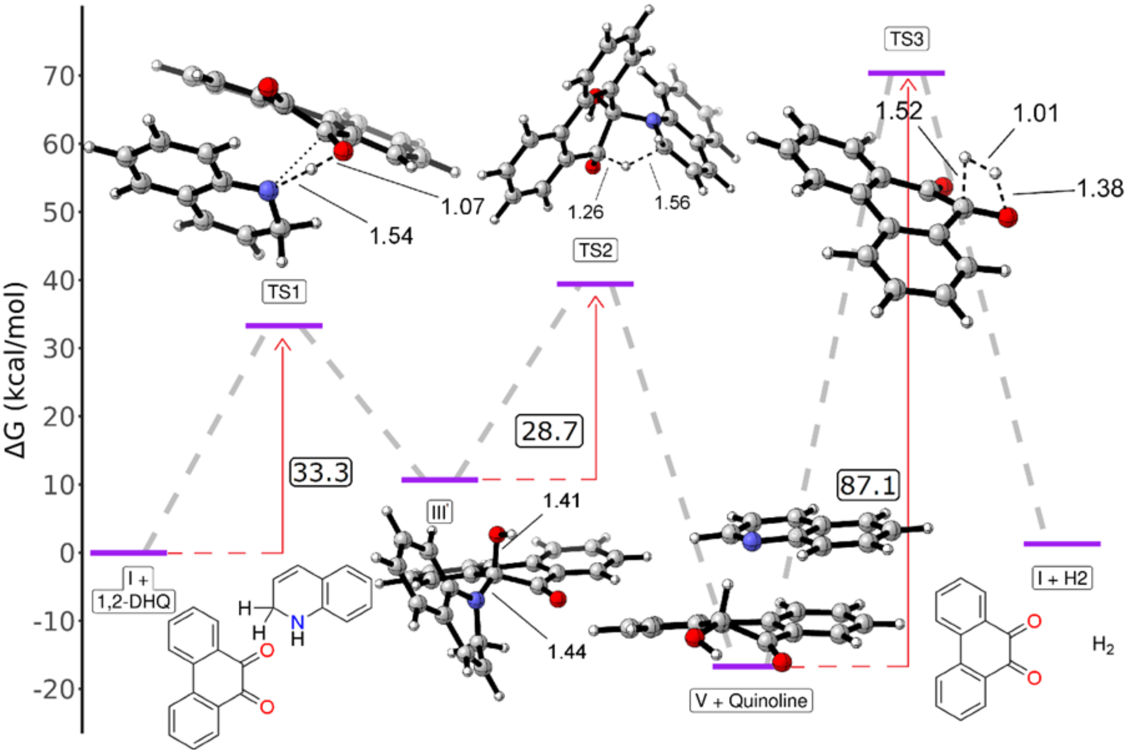

Figure S14 Potential Energy Surface (PES) of the second dehydrogenation mechanism using PBE0/def2-tzvp. Reaction intermediates labelled as in Scheme S2.

S13. 3 Third reaction mechanism: Epoxide mechanism

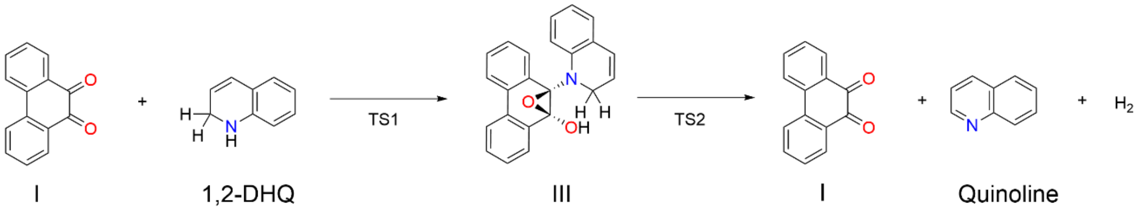

Scheme S3 Reaction mechanism of 1,2-DHQ dehydrogenation involving the epoxide intermediate (III), that yields quinoline and molecular hydrogen in a single concerted step.

The epoxide reaction mechanism is shown in Figures 8 and 9, and is the only pathway that can explain, with a relatively low free energy activation barrier, the formation of H_2 and quinoline, which are experimentally observed products. This mechanism was initially evaluated using the smaller 9,10-phenanthrenequinone cluster (Figure S15), and then expanded to the larger coronene-1,2-dione model (Section S11 and Figure 9), in order to provide a better representation of the bulk graphene catalyst. The PES of the reaction mechanism on the smaller cluster is shown in Figure S15.

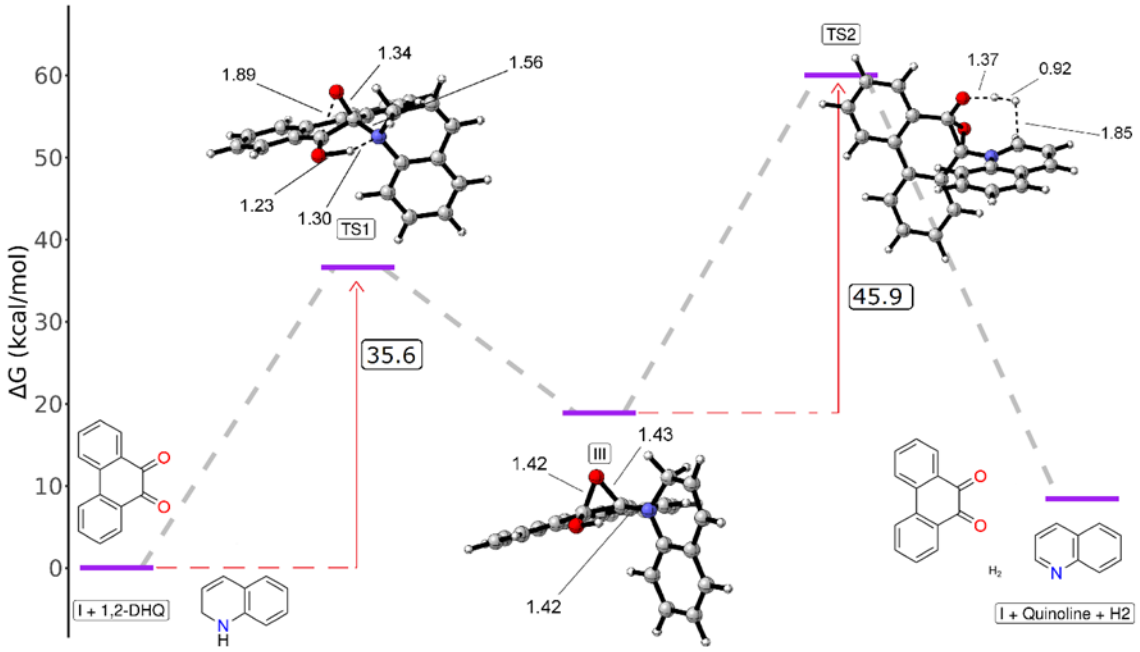

Figure S15 Potential Energy Surface of the epoxide intermediate mechanism for 1,2-DHQ dehydrogenation using PBE0/def2-tzvp. Reaction intermediates labelled as in Scheme S3 and Figure 8.

This epoxide mechanism begins with the concerted nucleophilic attack of 1,2-DHQ to a carbonyl carbon of the dione catalyst, while the N-H hydrogen is transferred to the other carbonyl group of the dione. This configuration leads to the simultaneous attack of the carbonyl oxygen on the contiguous carbonyl carbon, yielding the epoxide intermediate (III). From this intermediate, formation of H_2 takes place with the hydrogen at the 2-position of 1,2-DHQ and the hydrogen from the remaining OH group. As shown in Figure S15 (and Figure 9), the relatively high activation energy from this TS2 might be attributed to the C-H breaking energy, as the TS distance (1.85 Å) differs greatly from the intermediate III distance (1.1 Å).

S14. DFT density functional comparison.

To better compare the proposed reaction mechanism with the literature values of activation energies for similar carbocatalysts, the calculation of the energy barriers was carried out with other two DFT functionals, such as the hybrid M06-2X and the Generalized Gradient Approximation (GGA) PBE functional, which is among the most widely used in materials science and heterogeneous catalysis.

Table S6 Comparison of energies for dehydrogenation of the three reaction pathways at three different levels of theory. Free energies at 130 °C in kcal/mol, corresponding to Figures S13 (diol mechanism, section S13.1), Figure S14 (hemiaminal mechanism, section S13.2), and Figure S15 (epoxide mechanism, section S13.3).

		Reaction Mechanisms		
	Step	First diol	Second hemiaminal	Third epoxide
m06-2x-D3	TS1 (Barrier 1)	40.5	40.5	42.1
	Intermediate 1 [a,b]	10.5	10.5	14.7
	Barrier 2	48.6	36.5	52.3
	Intermediate 2 [a,b]	-25.9	-13.5	4.3
	Barrier 3	76.2	91.7	--
	Intermediate 3[c]	-2.8	3.1	--
PBE-D3	TS1 (Barrier 1)	25.1	25.1	34.0
	Intermediate 1 [a,b]	14.7	14.7	21.2
	Barrier 2	28.8	21.0	35.5
	Intermediate 2 [a,b]	-25.0	-12.7	-0.1
	Barrier 3	56.1	79.4	--
	Intermediate 3[c]	-7.3	-2.0	--
PBE0-D3	TS1 (Barrier 1)	33.3	33.3	35.6
	Intermediate 1 [a,b]	10.7	10.7	15.4
	Barrier 2	39.7	28.7	45.9
	Intermediate 2 [a,b]	-26.9	-15.4	2.3
	Barrier 3	69.0	87.1	--
	Intermediate 3[c]	-4.5	0.4	--

[a] Hemiaminal intermediate for first and second reactions (Schemes S1, S2, Figures S13-S14)

[b]Epoxide intermediate for third reaction (Scheme S3, Figure S15).

[c] Diketone (catalyst regeneration) and final hydrogen generation product for Schemes S1 and S2.

The reactivity trend is consistent within the *DFT* functionals tested (Table S6). For instance, hybrid functionals like M06-2x and PBE0 yield higher activation energies than PBE, but in turn, PBE offers less stabilization of the reaction intermediates.

While these mechanisms (S13.1 and S13.2) show relatively lower activation energies than the main reaction pathway in quinoline generation, they fail to accurately predict the formation of

molecular hydrogen with a low barrier, which is found experimentally in the reaction medium. They both show a free energy activation barrier larger than that for the proposed epoxide mechanism. Formation of hydrogen gas does probably follow the epoxide mechanism, as it is the path with the lower overall barrier, and agrees with experimental observations of the epoxide intermediate.

Although the barrier found in our reaction mechanism (45.9 kcal/mol) is relatively high, the value is similar as those found in previous studies of dehydrogenation transformations using different carbocatalysts. We have included a table summarizing previous results for comparative purposes (Table S7), using the same DFT functional for better comparison.

With another cluster (coronene), and including solvation effects, our barrier goes down from 45.9 to 41.1 kcal/mol (Table S5, PBE0-D3+SMD). With the same cluster (coronene) and PBE-D3 our free energy barrier still goes down to 35.2 kcal/mol (Table S5), which corresponds to an enthalpy barrier of 38.9 kcal/mol, in excellent agreement with similar computational studies in the literature (Table S7). For the sake of clarity, we compare the hydrogen-forming step activation energies, not the whole reaction mechanisms.

Overall, the values obtained for the reaction mechanisms at the PBE0 level of theory, while relatively high, suggest that further improvements in the methods and clusters may lead to lower barriers. In any case, the corresponding mechanism proposed (Figures 8, 9 and Section S13.3) make, in our opinion, chemical sense, and is in agreement with experiments which detect the presence of the epoxide and H_2 gas.

Table S7 Comparison of activation enthalpy barriers for dehydrogenation reactions using various heterogeneous catalysts.

Catalyst	Reactant	Software	DFT functional	ΔH^{bar} H_2 formation step (kcal/mol)	Ref
**Graphene**	Butane	CASTEP	PBE	71.7	[12]
**Graphene**	Ethylbenzene	VASP	PBE	59.7	[11]
**Nanodiamonds**	Ethylbenzene	VASP	PBE	33.4	[11]
**Graphene**	Tetrahydroquinoline	Gaussian	PBE	38.9	This work[a]
**Graphene**	Tetrahydroquinoline	Gaussian	PBE0	49.6	This work[b]

[a] Table S5, enthalpy from the ΔG=35.2 kcal/mol of Barrier 2, coronene.

[b] Table S5, enthalpy from the ΔG=45.6 kcal/mol of Barrier 2, coronene.

S15. Characterization of model molecules used as organocatalyst.

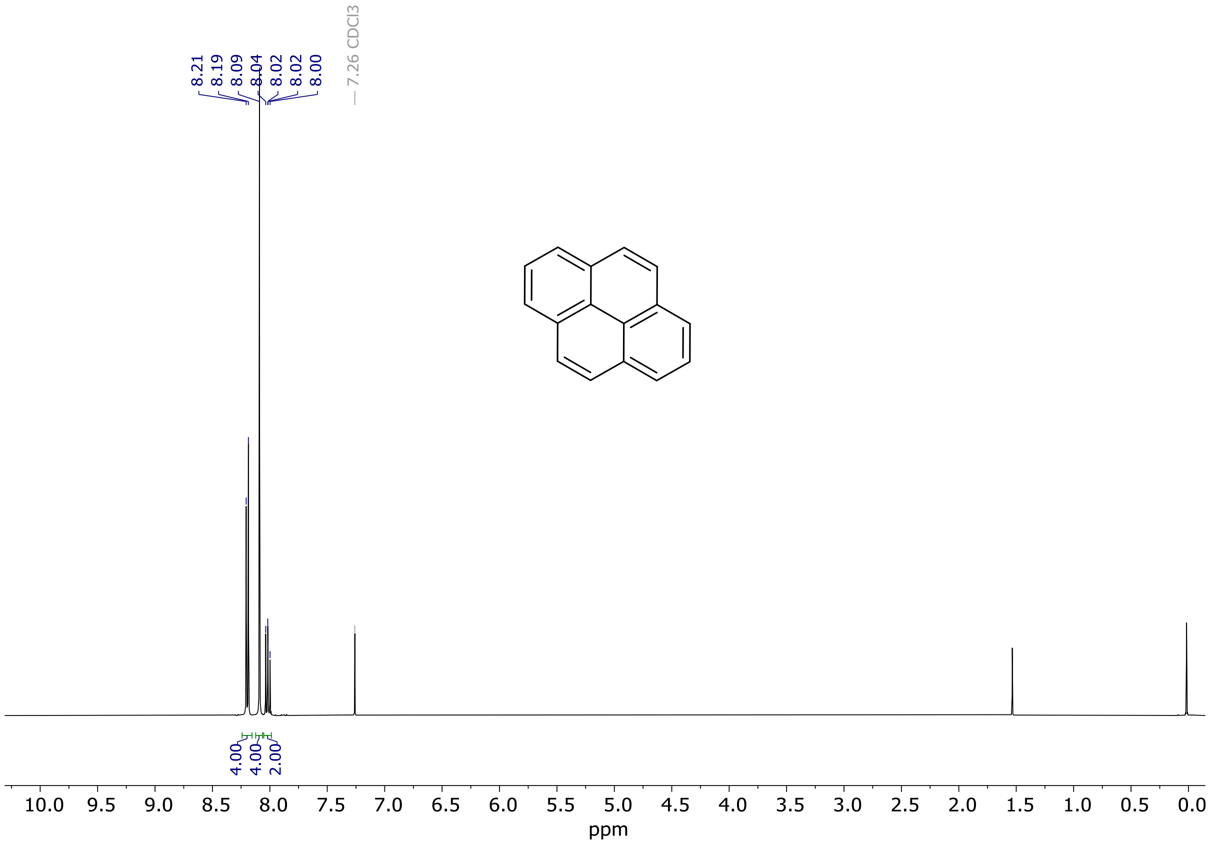

Figure S16 ^{1}H NMR spectrum (400 MHz) of model compound **A** in $CDCl_3$.

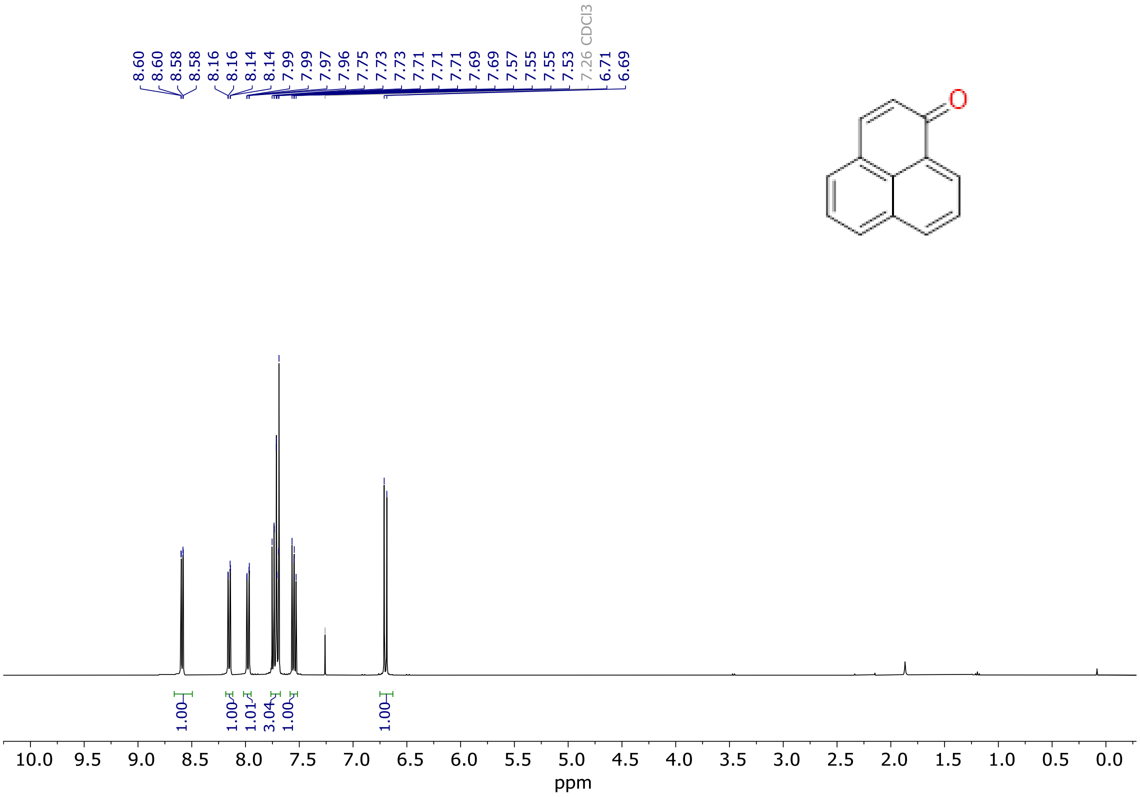

Figure S17 ^{1}H NMR spectrum (400 MHz) of model compound **B** in $CDCl_3$.

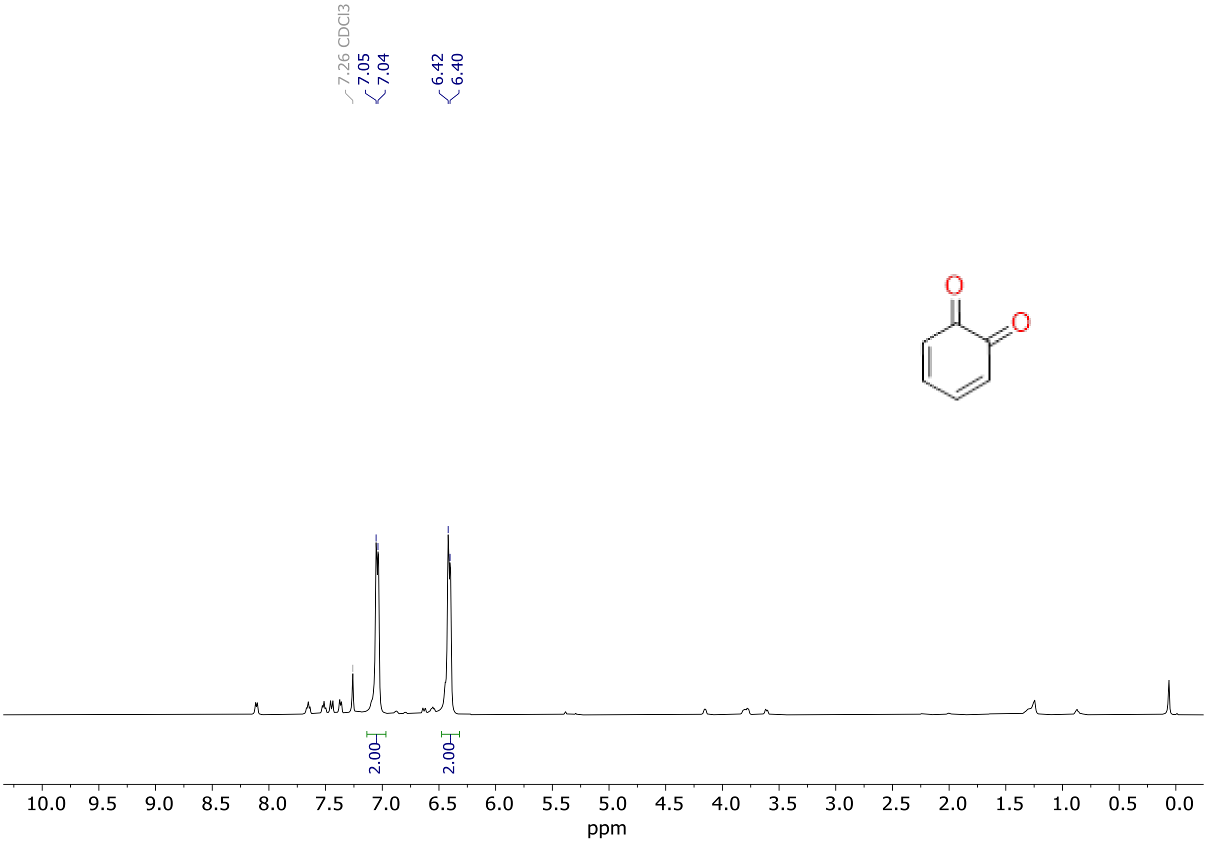

Figure S18 ^{1}H NMR spectrum (400 MHz) of model compound **C** in $CDCl_3$.

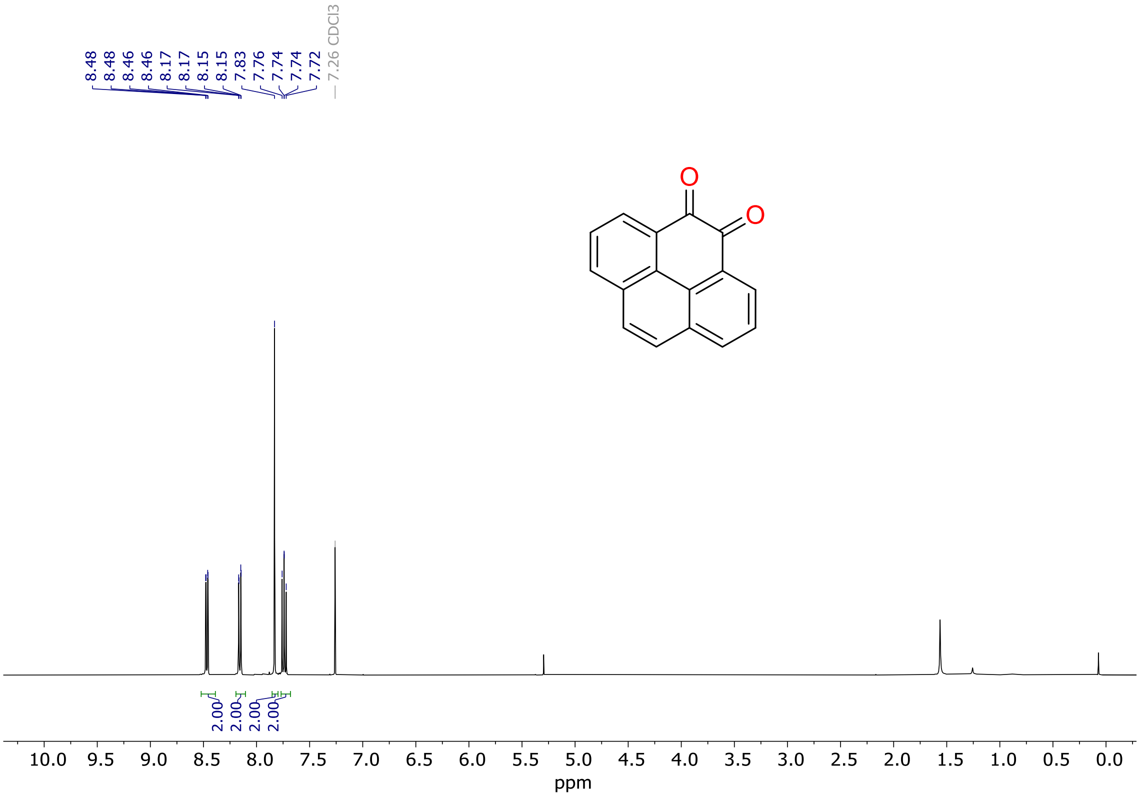

Figure S19 ^{1}H NMR spectrum (400 MHz) of model compound **D** in $CDCl_3$.

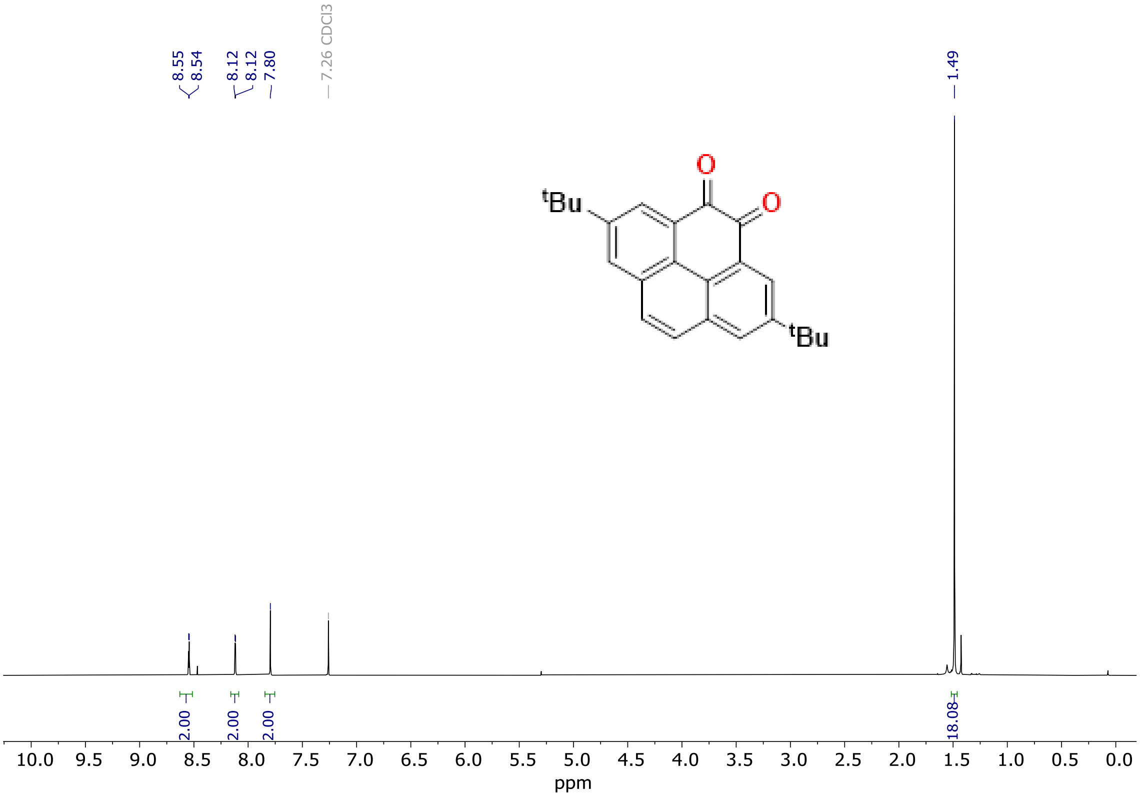

Figure S20 ^{1}H NMR spectrum (400 MHz) of model compound **E** in $CDCl_3$.

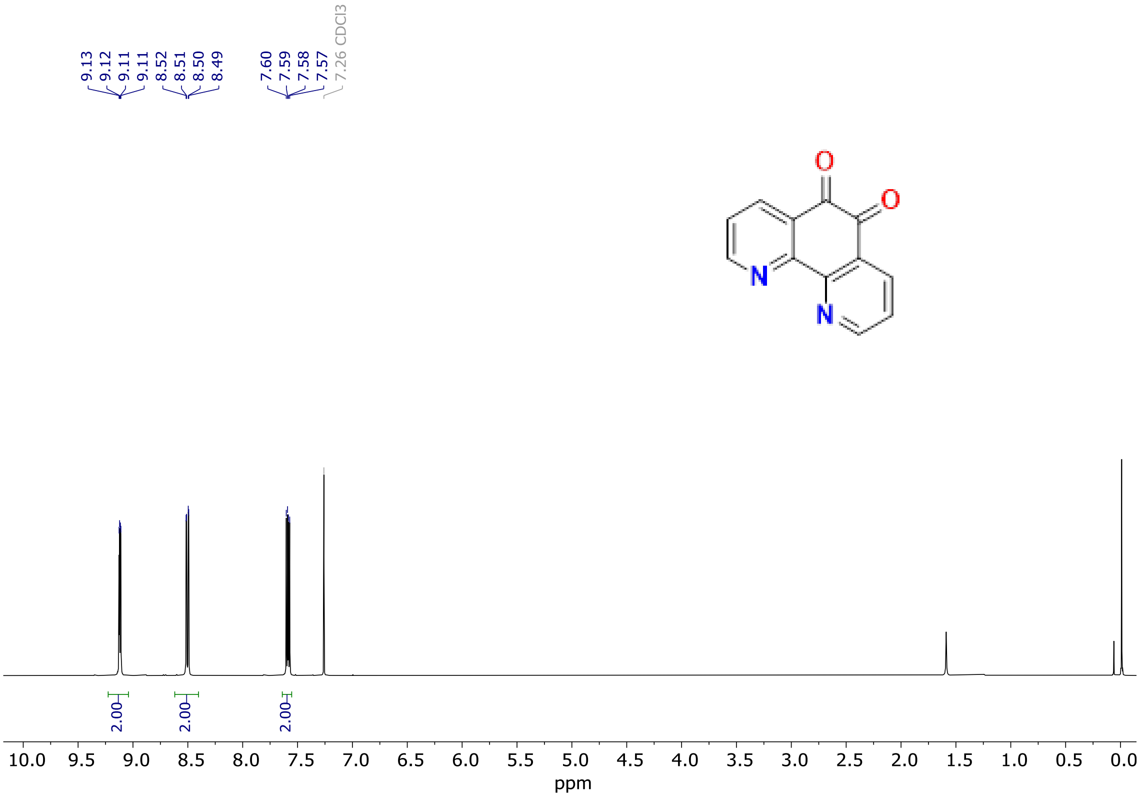

Figure S21 ^{1}H NMR spectrum (400 MHz) of model compound **F** in $CDCl_3$.

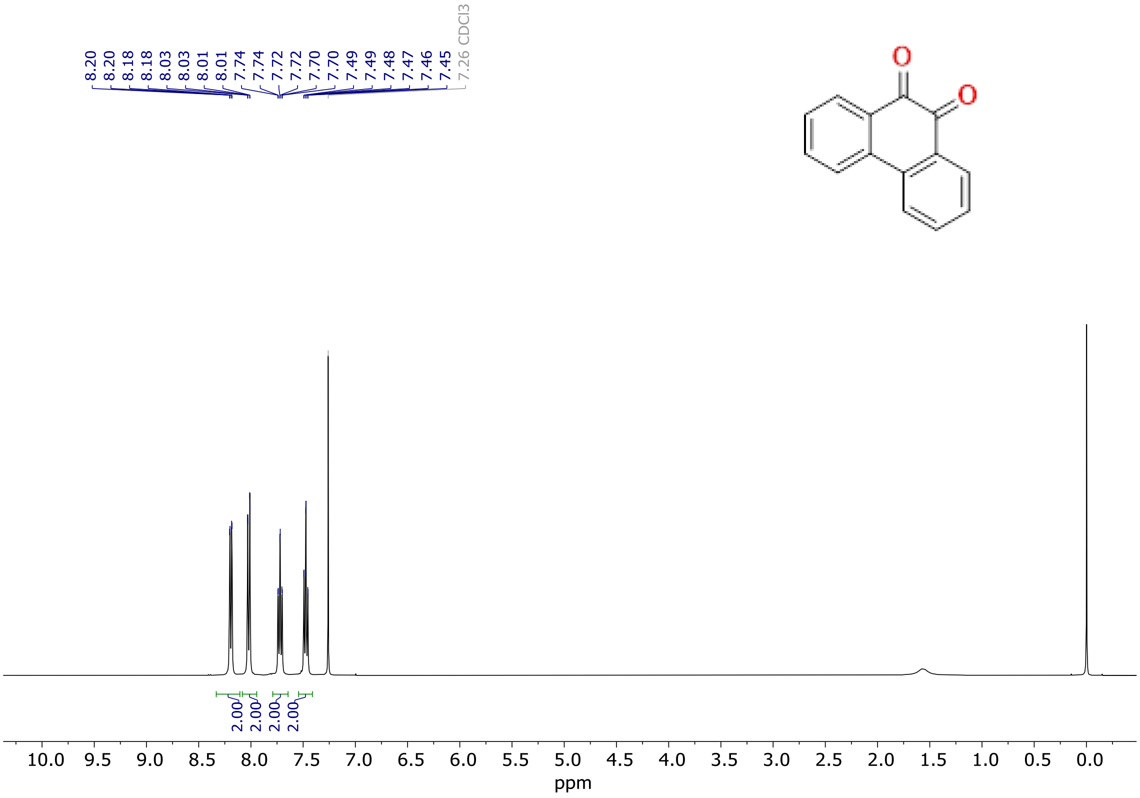

Figure S22 ^{1}H NMR spectrum (400 MHz) of model compound **G** in $CDCl_3$.

S16. References

(1) Collins, S. P.; Storrow, A.; Liu, D.; Jenkins, C. A.; Miller, K. F.; Kampe, C.; Butler, J. Synthesis and Characterization of 9,LO-Bis(Arylimino) -9,LO- Dihydrophenanthrenes, the Structure of (Z,Z)-9,10-Bis(Phenylimino)-9,10- Dihydrophenanthrene and PdCl 2- [(E,E) -9,10-Bis(Phenylimino) -9,LO- Dihydrophenanthrenel in the Solid State and in Solu. *Recueil des Trav. Chim. des Pays-Bas,* **2021**, *3*, 167–186.

(2) Zhao, Y.; Truhlar, D. G. The M06 Suite of Density Functionals for Main Group Thermochemistry, Thermochemical Kinetics, Noncovalent Interactions, Excited States, and Transition Elements: Two New Functionals and Systematic Testing of Four M06-Class Functionals and 12 Other Functionals. *Theor. Chem. Acc.,* **2008**, *120* (1–3), 215–241. https://doi.org/10.1007/s00214-007-0310-x.

(3) Chai, J.-D.; Head-Gordon, M. Long-Range Corrected Hybrid Density Functionals with Damped Atom–Atom Dispersion Corrections. *Phys. Chem. Chem. Phys.,* **2008**, *10* (44), 6615–6620. https://doi.org/10.1039/b810189b.

(4) Perdew, J. P.; Burke, K.; Ernzerhof, M. Generalized Gradient Approximation Made Simple. *Phys Rev Lett* **1996**, *77* (18), 3865–3868. https://doi.org/10.1103/PhysRevLett.77.3865.

(5) Adamo, C.; Barone, V. Toward Reliable Density Functional Methods without Adjustable Parameters: The PBE0 Model. *J. Chem. Phys.,* **1999**, *110* (13), 6158–6170. https://doi.org/10.1063/1.478522.

(6) Marenich, A. V.; Cramer, C. J.; Truhlar, D. G. Universal Solvation Model Based on Solute Electron Density and on a Continuum Model of the Solvent Defined by the Bulk Dielectric Constant and Atomic Surface Tensions. *J. Phys. Chem. B,* **2009**, *113* (18), 6378–6396. https://doi.org/10.1021/jp810292n.

(7) Grimme, S.; Antony, J.; Ehrlich, S.; Krieg, H. A Consistent and Accurate Ab Initio Parametrization of Density Functional Dispersion Correction (DFT-D) for the 94 Elements H-Pu. *J. Chem. Phys.,* **2010**, *132* (15), 154104. https://doi.org/10.1063/1.3382344.

(8) Weigend, F. Accurate Coulomb-Fitting Basis Sets for H to Rn. *Phys.Chem. Chem. Phys.,* **2006**, *8* (9), 1057–1065. https://doi.org/10.1039/b515623h.

(9) Bogdos, M. K.; Morandi, B. EveRplot: A Web-Based Shiny Application for Creating Energy vs Reaction Coordinate Diagrams. *J. Chem. Educ.,* **2023**, *100* (9), 3641–3644. https://doi.org/10.1021/acs.jchemed.3c00319.

(10) Legault, C. Y. CYLview20. http://www.cylview.org: Université de Sherbrooke October 31, 2020.

(11) Feng, L.; Ali, S.; Xu, C.; Cao, S.; Tuci, G.; Giambastiani, G.; Pham-Huu, C.; Liu, Y. Assessing the Nature of Active Sites on Nanodiamonds as Metal-Free Catalysts for the EB-to-ST Direct Dehydrogenation Using a Catalytic Approach. *ACS Catal.,* **2022**, *12* (10), 6119–6131. https://doi.org/10.1021/acscatal.2c00825.

(12) Brooks, A.; Jenkins, S. J.; Wrabetz, S.; McGregor, J.; Sacchi, M. The Dehydrogenation of Butane on Metal-Free Graphene. *J Colloid Interface Sci* **2022**, *619*, 377–387. https://doi.org/10.1016/j.jcis.2022.03.128.
